# Predictive Capability of Dual Trajectories of Central Adiposity Indices Combined With Glucose for Cardiovascular Diseases

**DOI:** 10.1111/1753-0407.70081

**Published:** 2025-04-25

**Authors:** Gangjiao Zhu, Xiong Ding, Hui Zhou, Janis M. Nolde, Thomas Beaney, Dan Wu, Yulong Lan, Yan Tian, Ruolin Zhang, Bolu Yang, Shuohua Chen, Bifeng Yuan, Shouling Wu, Lijing L. Yan

**Affiliations:** ^1^ School of Public Health Wuhan University Wuhan China; ^2^ Global Heath Research Center Duke Kunshan University Kunshan China; ^3^ Xiangya School of Nursing Central South University Changsha China; ^4^ Department of Nephrology University Medical Centre Freiburg Freiburg Germany; ^5^ School of Public Health Imperial College London London UK; ^6^ Second Affiliated Hospital of Shantou University Medical College Shantou China; ^7^ School of Public Health North China University of Science and Technology Tangshan China; ^8^ Harvard T.H. Chan School of Public Health Harvard University Boston USA; ^9^ Department of Cardiology Kailuan General Hospital Tangshan China

**Keywords:** cardiovascular diseases, central adiposity, dual trajectories, fasting plasma glucose

## Abstract

**Background:**

This aimed to quantify the association between dual trajectory patterns combining seven central adiposity (CA) indices and fasting plasma glucose (FPG) with cardiovascular disease (CVD) risk in adults, and to compare their predictive performance.

**Methods:**

The Kailuan Study, a prospective study initiated in June 2006, included 39 772 adults without pre‐existing CVD as of 2010. Dual trajectories of seven CA indices combined with FPG were recorded from 2006 to 2010 to predict CVD risk from 2010 to 2021. Cox regression models were used to estimate hazard ratios (HRs) and 95% confidence intervals (CIs) for incident CVD.

**Results:**

During a median follow‐up of 11.0 years, 2715 incident CVD events were recorded. Four distinct patterns of CA indices (waist circumference, waist‐to‐height ratio, abdominal volume index, body roundness index) and three distinct patterns of other CA indices (waist‐to‐hip ratio, conicity index, A body shape index) combined with FPG were identified. Compared with the lowest‐risk group, the highest‐risk group exhibited a significantly higher CVD risk (adjusted HRs [95% CIs]: 2.41 [2.02–2.86], 2.57 [2.18–3.05], 2.25 [1.92–2.63], 2.35 [2.01–2.73], 2.08 [1.74–2.49], 1.97 [1.72–2.26], 1.81 [1.58–2.07], respectively). Overall, the predictive capabilities were generally similar, with the combination of waist circumference and FPG showing a slightly better predictive performance compared with other patterns.

**Conclusions:**

Distinct patterns of dual trajectories involving seven CA indices combined with FPG were associated with CVD risk. The results suggest that the combination of waist circumference and FPG may have greater clinical significance in predicting CVD risk.

## Graphical abstract

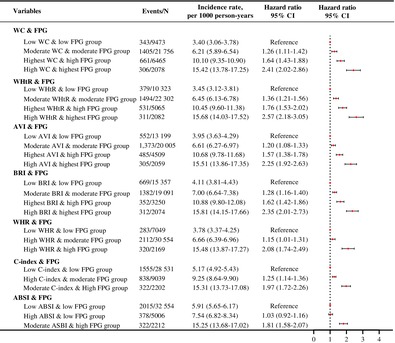

**Summary**

- Distinct patterns of seven CA indices along with FPG were associated with CVD risk.
- Other groups showed higher CVD risk than all low CA indices and low FPG groups.
- FPG coincided with increased adiposity indices.
- Higher FPG level was significantly associated with a subsequent elevated CVD risk.

## 1 Introduction

Cardiovascular diseases (CVD) remain the leading cause of premature mortality and disability worldwide [1]. From 1990 to 2019, the CVD prevalence nearly doubled from 271 million to 523 million, while CVD‐related deaths increased from 12.1 million to 18.6 million [1]. Despite ongoing preventive efforts, global trends indicate a significant rise in disability‐adjusted life years and years of life lost due to CVD [2], highlighting the need for advanced research into the risk factors contributing to this global health burden.

High fasting plasma glucose (FPG) level and adiposity stand out as critical threats to cardiovascular health [3, 4]. The escalating prevalence of diabetes, alongside the obesity epidemic, suggests a potential interaction between hyperglycemia and adiposity. However, the co‐evolution pattern of obesity and diabetes, especially their combined effects on CVD, remains underexplored.

Traditional methods for assessing obesity may not reliably predict CVD risk. For instance, the inability of body mass index (BMI) to differentiate between lean body mass and fat mass may compromise the accuracy of assessing obesity and its associated CVD risk [5, 6]. Recent studies indicated that fat distribution, rather than overall adiposity, is a significant determinant of CVD risk [7, 8, 9]. Notably, waist circumference (WC), an indicator of central adiposity (CA), is an important predictor of CVD [10]. Substantial evidence supports the association between various CA indices and the occurrence of CVD events [9, 11, 12, 13]. Studies have consistently demonstrated that glucose metabolism and cardiometabolic measures (CA), including waist circumference (WC) [14], waist‐to‐hip ratio (WHR) [15], body roundness index (BRI) [16], and fasting plasma glucose (FPG) [17, 18], are key risk factors for CVD. The interaction between CA and glucose metabolism may strengthen their combined predictive value for CVD. Compared with relying solely on a single baseline measurement, longitudinal studies provide comprehensive insights by accumulating repeated measurement data, while they have mainly concentrated on individual trajectories rather than combined trajectories [14, 15, 16, 17]. In contrast to individual trajectories, group‐based trajectory modeling identifies clusters of individuals with similar patterns across multiple indicators [19], capturing the joint progression of interrelated measurements.

This study aimed to identify the longitudinal patterns of seven CA indices combined with FPG level to clarify their association with incident CVD risk, thereby supporting targeted preventive strategies. Using data from the Kailuan Study, a prospective cohort in China, the relationship between dual trajectories of the seven CA indices and FPG level with incident CVD was first assessed, integrating longitudinal data on adiposity and glucose control. The predictive performance of these combined indices for the study outcomes was thereafter compared.

## 2 Materials and Methods

### 2.1 Study Design and Population

The Kailuan Study (Registration No. CHICTR‐TNRC‐11001489) is an ongoing community‐based prospective cohort in Tangshan, China, aimed at examining risk factors for chronic non‐communicable diseases. This study was approved by the Ethics Committee of the Kailuan General Hospital [20]. All participants provided written informed consent. This study followed the Strengthening the Reporting of Observational Studies in Epidemiology (STROBE) guidelines.

**FIGURE 1:**
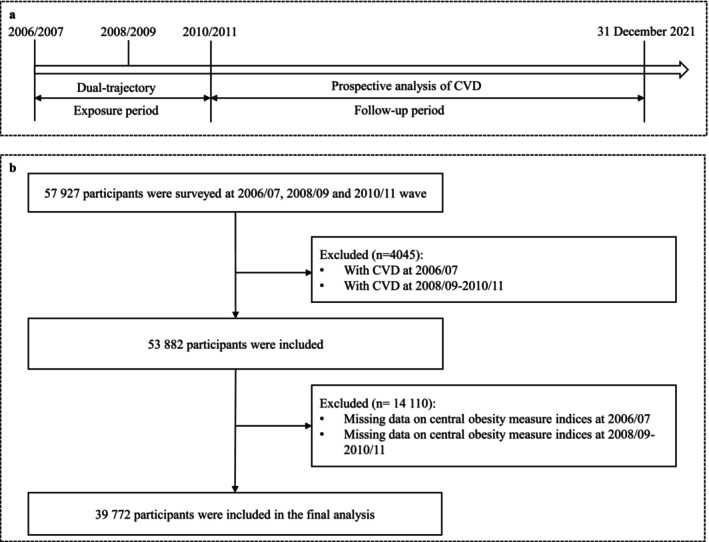
The flowchart of the participant selection process.

Detailed information about the study design and procedures has been previously reported [21]. In brief, between June 2006 and October 2007, 101 510 adults aged 18–98 years old, including 81 110 men and 20 400 women, were recruited from the Kailuan community and underwent biennial health exams. To establish the dual trajectories of seven CA indexes combined with FPG, individuals who had not been diagnosed with CVD by 2010 and had complete data on seven CA indices and FPG across three consecutive waves (2006–2007, 2008–2009, and 2010–2011) were included in the final analysis, totaling 39 772 participants. Figure 1 illustrates the inclusion and exclusion criteria.

### 2.2 Ascertainment of Exposures

The exposure variables were dual trajectory patterns of seven CA indices combined with FPG, which were identified using a group‐based multi‐trajectory model with data from three consecutive health surveys. Specifically, seven distinct CA indices were considered: WC, WHR, waist‐to‐height ratio (WHtR), conicity index (C‐index), A body shape index (ABSI), abdominal volume index (AVI), and body roundness index (BRI). Dual trajectories were modeled for each of the seven CA indices combined with FPG, resulting in seven sets of trajectories. Anthropometric measurements, including height, weight, WC, and hip circumference, were collected by trained staff following standardized procedures. Weight was measured to the nearest 0.1 kg while participants wore lightweight clothing without shoes. Height was measured to the nearest 0.1 cm using a stadiometer. WC was measured at the midpoint between the lower rib and the iliac crest, and hip circumference at the widest point of the buttocks, both to the nearest 0.1 cm using a non‐elastic tape. Calculations of the CA indices are detailed in Table S1. FPG level was determined using the hexokinase/glucose‐6‐phosphate dehydrogenase method with an automatic analyzer (Hitachi 747), in which blood samples were collected after 8–12 h of overnight fasting and tested within 4 h.

### 2.3 Ascertainment of Outcomes

The outcome of interest was incident CVD. Consistent with previous studies [22], incident CVD was assessed annually during follow‐up. CVD cases were identified using ICD‐10 codes (I60, I61, and I63 for stroke; I21 for myocardial infarction). Potential CVD events were tracked through four sources: (1) the Municipal Social Insurance Institution, covering all participants; (2) Hospital Discharge Register centers; (3) death certificates; and (4) biennial questionnaire surveys. All participants were monitored from the end of the exposure period until the date of CVD diagnosis, death, or December 31, 2021, whichever occurred first.

### 2.4 Assessment of Potential Covariates

Potential covariates, such as age, sex, marital status, educational level, smoking and drinking status, physical activity, salt intake habits, and medical history were collected via questionnaires administered during the 2010–2011 period. Educational level was divided into illiteracy/primary school, middle school, and vocational/high school or above. Smoking was defined as smoking more than one cigarette per day in the last year, and drinking status was based on self‐reported alcohol consumption in the past year [21]. Physical activity was classified as none, occasional (< 80 min/week), and active (≥ 80 min/week) [23]. Habitual salt intake was classified as low (< 6 g/day), medium (6–10 g/day), and high (> 10 g/day) [24].

BMI was calculated as weight (kg) divided by the height squared (m^2^). Hypertension was defined as systolic blood pressure ≥ 140 mm Hg, or/and diastolic blood pressure ≥ 90 mm Hg, a self‐reported history of hypertension, or any use of antihypertensive drugs. Diabetes was defined as FPG concentration ≥ 7.0 mmol/L, a self‐reported history of diabetes, or any use of hypoglycemic medications. The estimated glomerular filtration rate (eGFR) was determined using the creatinine equation from the Chronic Kidney Disease Epidemiology Collaboration [25]. Laboratory analyses of biochemical indicators, including low‐density lipoprotein cholesterol, high‐density lipoprotein cholesterol, high‐sensitivity C‐reactive protein, and creatinine, were performed by an auto‐analyzer (Hitachi 747) at the central laboratory of Kailuan Hospital.

### 2.5 Missing Data Imputation

To test the stability of the findings, sensitivity analysis was conducted using multiple imputation for missing covariates, yielding results consistent with the primary analysis. To preserve the integrity of the data structure, the fully conditional specification (FCS) was employed. Discriminant analysis was applied to categorical variables to ensure that imputed values remained within their original categories. For continuous variables, linear regression was used to generate imputed values that closely aligned with the variables' underlying distribution patterns.

### 2.6 Statistical Analysis

Participants' baseline characteristics were presented as mean and standard deviation (SD) or median (25–75th percentiles) for normally distributed continuous variables, median and interquartile range (IQR) for skewed continuous variables, and frequency and percentage for categorical variables. Differences between groups were assessed using independent t test and one‐way analysis of variance (ANOVA) for normally distributed continuous variables, Kruskal–Wallis test for skewed continuous variables, and Chi‐square (*χ* ^2^) test for categorical variables.

The group‐based multi‐trajectory modeling (PROC TRAJ SAS plug‐in) was employed to identify dual trajectories of seven CA indices combined with FPG [19, 26]. Consistent with the previous approach [27], a model comprising two trajectory groups was initially constructed and then expanded to three, four, and five trajectory groups. For each number of trajectory groups, polynomial orders (e.g., quadratic and linear specifications) for each trajectory shape were tested to identify the optimal fitting model. The final number of dual trajectories was determined based on the following criteria [26, 28]: (1) Sufficient sample size in each multi‐trajectory group (> 5% of the sample); (2) Best‐fit model based on the lowest Bayesian information criterion; (3) Logged Bayes factor approximating 2∆BIC (a value above 6 is recommended); (4) Average posterior probability values > 0.7 in each group to assign membership, indicating satisfactory internal reliability.

Incidence rates were calculated as events per 1000 person‐years. The Kaplan–Meier method was used to estimate the cumulative incidence of CVD for each trajectory group, and intergroup comparisons were made using the log‐rank test. The Cox proportional‐hazards regression model was utilized to calculate hazard ratios (HRs) and 95% confidence intervals (CIs) for the risk of developing CVD across the dual trajectories of seven CA indices combined with FPG. The proportional‐hazards assumption was evaluated and satisfied. C statistics, the net reclassification index (NRI), and integrated discrimination improvement (IDI) were used to compare the predictive performances of the seven CA indices combined with FPG for CVD. Adjustments were made for potential confounders, including age, sex, educational level, marital status, smoking status, drinking status, habitual salt intake, physical activity, low‐density lipoprotein cholesterol, high‐density lipoprotein cholesterol, ln high‐sensitivity C‐reactive protein, ln eGFR, hypertension, and the use of antihypertensive, antidiabetic, and lipid‐lowering medications.

Several sensitivity analyses were conducted to examine the robustness of the findings. First, additional adjustments for BMI were made to assess whether BMI could confound the associations. Second, adjustments were made for baseline single measurements of the distinct CA indices combined with FPG (2006–2007 and 2010–2011). Third, to minimize potential bias arising from missing data, individuals with missing potential covariates were excluded from the main analysis. However, to assess the robustness of the findings, an additional sensitivity analysis was carried out, in which multiple imputation was applied to fill in missing covariate data. The imputation process employed FCS using linear regression for continuous variables and discriminant analysis for categorical variables, generating 1 imputed dataset. The main and sensitivity analyses were compared to assess whether missing data could influence the conclusions. Fourth, to account for the potential influence of drug treatments on the outcomes, re‐analysis was conducted excluding participants who had taken antihypertensive, hypoglycemic, or lipid‐lowering medications. To mitigate the risk of reverse causality, participants with outcome events occurring within the first two years of follow‐up were excluded.

Between April 2023 and September 2024, all statistical analyses were performed using SAS 9.4 software (SAS Institute Inc., Cary, NC, USA). A two‐sided *p* < 0.05 was considered statistically significant.

## 3 Results

**FIGURE 2:**
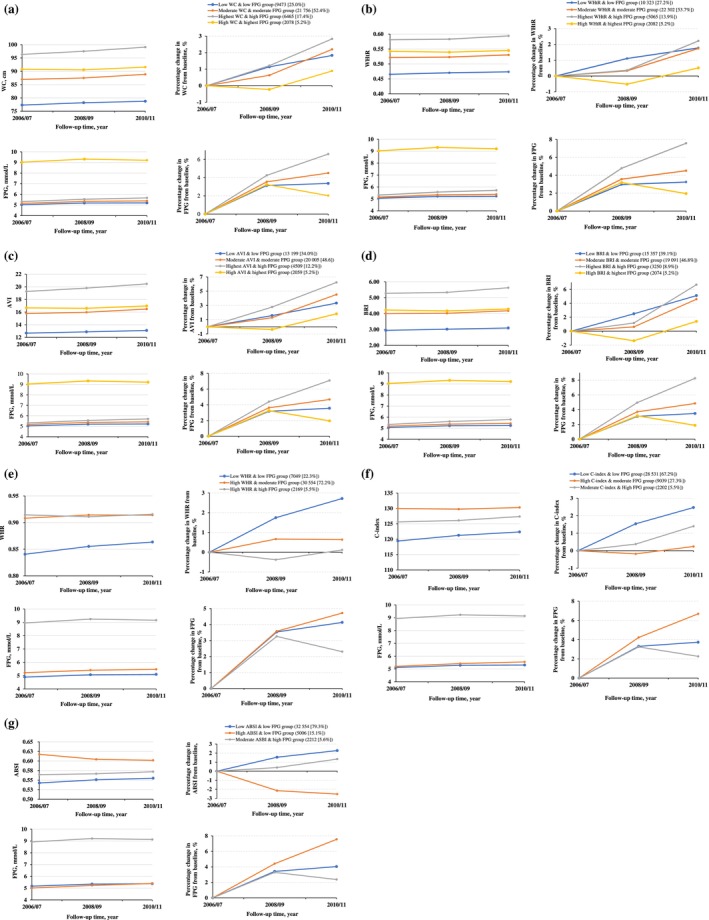
Dual trajectory groups of different CA indices and FPG using group‐based multi‐trajectory modeling. Dots show group‐specific mean observed levels (or percentage change from baseline) while solid lines represent the best fitted trajectories. Different CA indices and FPG were modeled as a function of follow‐up time. ABSI, a new body shape index; AVI, abdominal volume index; BRI, body roundness index; CA, Central adiposity; C‐index, conicity index; FPG, fasting plasma glucose; WC, waist circumference; WHR, waist‐to‐hip ratio; WHtR, waist‐to‐height ratio.

A total of 39 772 adults were included in the study after excluding individuals with missing key covariates to ensure robustness. The mean (SD) age at baseline was 52.6 (11.6) years, and 31 471 (79.1%) were male. The results indicated that three or four trajectory patterns optimally represent the dual trajectories of seven CA indices (WC, WHtR, AVI, BRI, WHR, C‐index, and ABSI) combined with FPG, based on interpretability, model adequacy criteria, and simplicity (Figure 2). Participants with initially high corpulence tended to maintain this trajectory over time. The model adequacy criteria are detailed in Table S2. As illustrated in Figure 2a–d, four distinct patterns were identified for the CA indices (WC, WHtR, AVI, and BRI) combined with FPG from 2006 to 2010: (1) low CA indices & low FPG, (2) moderate CA indices & moderate FPG, (3) highest CA indices & high FPG, and (4) high CA indices & highest FPG. In contrast, for WHR, C‐index, and ABSI combined with FPG, three distinct trajectory patterns were identified from 2006 to 2010 (Figure 2e–g): (1) low CA indices & low FPG, (2) high CA indices & low/moderate FPG, and (3) moderate/high CA indices & high FPG. Overall, each trajectory pattern exhibited an increasing trend versus the 2006 baseline.

To test the stability of the findings, sensitivity analysis was conducted using multiple imputation for missing covariates, yielding results consistent with the primary analysis. The findings before and after multiple imputation are presented in Table S3. For instance, LDL‐C, the most frequently missing variable, had 404 missing values, accounting for less than 1% of the dataset, as detailed in Table S3. Detailed characteristics of individuals within the distinct patterns of seven CA indices combined with FPG are presented in Tables S4–S10.

During a median follow‐up of 11.0 (range, 10.6–11.3) years, 2715 incident CVD events were recorded (Table 1). Generally, cases experiencing CVD had higher rates of hypertension and diabetes, along with advanced age, male gender, lower educational level, elevated systolic blood pressure, diastolic blood pressure, high‐sensitivity C‐reactive protein level, and medication usage.

**TABLE 1:** The study participants baseline characteristics according to with and without events incident during 2010–2021 Follow‐Up Period.

Characteristics	Total	Without events	With CVD events	*p*
	(*n* = 39 772)	(*n* = 37 057)	(*n* = 2715)	
Age, mean (SD), y	52.6 (11.6)	52.2 (11.6)	58.4 (10.1)	< 0.001
Male	31 471 (79.1)	29 062 (78.4)	2409 (88.7)	< 0.001
Education background				< 0.001
Illiterate/elementary	2808 (7.0)	2503 (6.7)	305 (11.2)	
Middle school	27 433 (69.0)	25 383 (68.5)	2050 (75.5)	
Vocational/high school above	9531 (24.0)	9171 (24.8)	360 (13.3)	
Married or remarriage	39 299 (98.8)	36 605 (98.8)	2694 (99.2)	0.11
Current smoking	14 004 (35.2)	12 973 (35.0)	1031 (38.0)	0.008
Current drinking	14 183 (35.7)	13 231 (35.7)	952 (35.1)	0.80
Physical activities				0.09
No	12 420 (31.2)	11 620 (31.4)	800 (29.5)	
Occasionally	22 014 (55.4)	20 505 (55.3)	1509 (55.6)	
Frequently	5338 (13.4)	4932 (13.3)	406 (14.9)	
Salt intake habits				
High	4198 (10.6)	3867 (10.4)	331 (12.2)	0.07
Medium	28 635 (72.0)	26 702 (72.1)	1933 (71.2)	
Low	6939 (17.4)	6488 (17.5)	451 (16.6)	
BMI, mean (SD), kg/m^2^	25.1 (3.2)	25.0 (3.2)	25.7 (3.2)	< 0.001
SBP, mean (SD), mm Hg	130.6 (18.2)	129.9 (18.0)	140.3 (19.1)	< 0.001
DBP, mean (SD), mm Hg	84.4 (10.3)	84.1 (10.2)	88.4 (10.7)	< 0.001
LDL‐C, mean (SD), mmol/L	2.6 (0.8)	2.6 (0.7)	2.9 (0.8)	< 0.001
HDL‐C, mean (SD), mmol/L	1.5 (0.4)	1.5 (0.4)	1.5 (0.4)	< 0.001
Hs‐CRP, median (IQR), mg/L	1.0 (0.5–2.5)	1.00 (0.4–2.4)	1.2 (0.6–3.0)	< 0.001
eGFR, median (IQR), mL/min/1.73 m^3^	90.9 (75.0–103.4)	91.2 (75.3–103.7)	86.3 (71.9–98.5)	< 0.001
Hypertension	18 962 (47.7)	17 063 (46.0)	1899 (69.9)	< 0.001
Diabetes mellitus	4242 (10.7)	3710 (10.0)	532 (19.6)	< 0.001
Medication use				
Antihypertensive	4035 (10.1)	3544 (9.6)	491 (18.1)	< 0.001
Antidiabetic	1152 (2.9)	998 (2.7)	154 (5.7)	< 0.001
Lipid‐lowering	326 (0.8)	295 (0.8)	31 (1.1)	0.16

*Note:* Data are expressed as mean (SD) for normally distributed continuous variables. *p* values indicate statistical significance for differences between participants with and without CVD events.

Abbreviations: BMI, body mass index; CVD, cardiovascular disease; DBP, diastolic blood pressure; eGFR, estimated glomerular filtration rate; HDL‐C, high‐density lipoprotein cholesterol; Hs‐CRP, high‐sensitivity C‐reactive protein; LDL‐C, low‐density lipoprotein cholesterol; SBP, systolic blood pressure.

Kaplan–Meier curves indicated that individuals in the other groups had a higher risk of CVD events compared with those in the low CA indices & low FPG group throughout the follow‐up period (Figures S1–S7; log‐rank test, *p* < 0.001).

**FIGURE 3:**
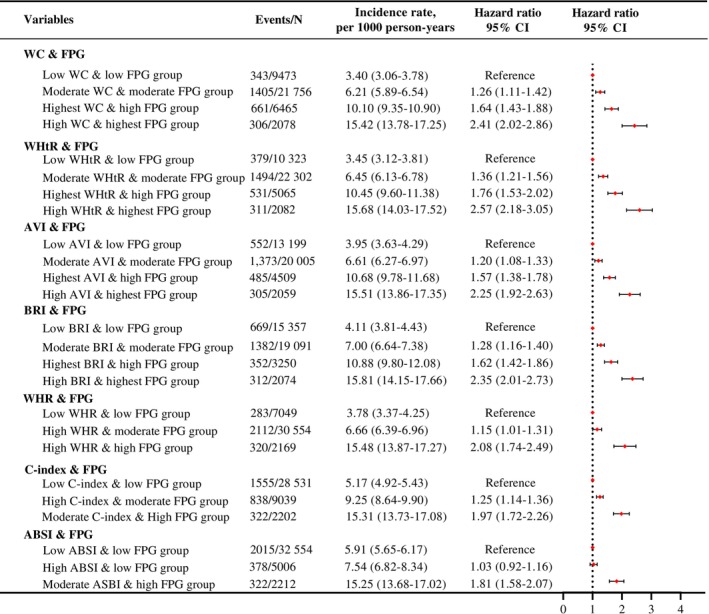
Associations between dual trajectory groups of different CA indices and FPG with incident cardiovascular diseases. Adjusted for age, sex, education background, marital status, smoking status, drinking status, physical activities, salt intake habits, LDL‐C, HDL‐C, ln eGFR, ln CRP, hypertension, diabetes, using antihypertensive, using antidiabetic, using lipid‐lowering. ABSI, a new body shape index; AVI, abdominal volume index; BRI, body roundness index; CA, Central adiposity; C‐index, conicity index; FPG, fasting plasma glucose; WC, waist circumference; WHR, waist‐to‐hip ratio; WHtR, waist‐to‐height ratio.

Compared with all low CA indices and low FPG groups, other groups exhibited a significant and sustained increase in CVD risk, as shown in Figure 3. Among the four distinct patterns, the high CA and highest FPG group had a higher CVD risk compared with the low CA and low FPG group for WC, WHtR, AVI, and BRI combined with FPG (HRs and 95% CIs: 2.41 [2.02–2.86], 2.57 [2.18–3.05], 2.25 [1.92–2.63] and 2.35 [2.01–2.73], respectively), after adjusting for confounders. Regarding three distinct patterns, the moderate/high CA and high FPG group had a higher CVD risk compared with the low CA and low FPG group for WHR, C‐index, and ABSI combined with FPG (adjusted HRs and 95% CIs: 2.08 [1.74–2.49], 1.97 [1.72–2.26], and 1.81 [1.58–2.07], respectively).

Table 2 shows that the distinct patterns of seven CA indices along with FPG display strong predictive capabilities for CVD, all with C‐statistics exceeding 70%. After adjusting for potential confounders, the NRI analysis suggested that the dual trajectory patterns of WHtR combined with FPG (NRI and 95% CI: 24.20 [20.62–27.78]) and BRI combined with FPG (NRI and 95% CI: 10.04 [6.37 to 13.72]) had stronger predictive abilities compared to WC combined with FPG. However, dual trajectory patterns of other CA indices combined with FPG either had weaker predictive abilities or showed no statistically significant difference compared with the dual trajectory patterns of WC combined with FPG.

**TABLE 2:** Reclassification and discrimination statistics for predicting cardiovascular disease by dual trajectory groups of different CA indices and FPG.

Patterns of seven CA indices combined with FPG	C statistics, % estimate (95% CI)	Difference C statistics, % estimate (95% CI)	*p*	Continuous IDI, % estimate (95% CI)	*p*	Continuous NRI, % estimate (95% CI)	*p*
WC & FPG	71.19 (70.10–72.27)	Reference		Reference		Reference	
WHtR & FPG	71.38 (70.38–72.38)	0.19 (−0.08 to 0.47)	0.16	0.01 (−0.01 to 0.04)	0.38	24.20 (20.62 to 27.78)	< 0.001
AVI & FPG	71.17 (70.10–72.24)	−0.02 (−0.14 to 0.11)	0.81	0 (−0.02 to 0.02)*	0.89	−1.42 (−4.30 to 1.45)	0.47
BRI & FPG	71.36 (70.35–72.38)	0.18 (−0.13 to 0.48)	0.25	0 (−0.03 to 0.03)*	0.83	10.04 (6.37 to 13.72)	< 0.001
WHR & FPG	70.99 (69.92–72.06)	−0.20 (−0.42 to 0.02)	0.08	−0.07 (−0.10 to −0.04)	< 0.001	−10.34 (−14.17 to −6.51)	< 0.001
C‐index & FPG	70.98 (69.87–72.09)	−0.20 (−0.39 to −0.02)	0.034	−0.04 (−0.07 to −0.01)	0.006	−12.90 (−16.61 to −9.18)	< 0.001
ABSI & FPG	70.89 (69.78–71.99)	−0.30 (−0.52 to −0.08)	0.008	−0.09 (−0.13 to −0.06)	< 0.001	−5.32 (−9.21 to −1.44)	0.007

*Note:* Adjusted for age, sex, education background, marital status, smoking status, drinking status, physical activities, salt intake habits, LDL‐C, HDL‐C, ln eGFR, ln CRP, hypertension, diabetes, using antihypertensive medications, using antidiabetic medications, using lipid‐lowering medications.

Abbreviations: ABSI, a new body shape index; AVI, abdominal volume index; BRI, body roundness index; CA, central adiposity; C‐index, conicity index; FPG, fasting plasma glucose; IDI, Integrated Discrimination Improvement; NRI, Net Reclassification Improvement; WC, waist circumference; WHR, waist‐to‐hip ratio; WHtR, waist‐to‐height ratio.

* 0.01% < IDI.

The results remained robust following additional adjustments for BMI and various time‐independent levels of CA indices combined with FPG in 2006–2007 or 2010–2011 (Table S11). Moreover, these results were consistent both before and after the multiple imputations of missing covariates, as well as after excluding individuals using medications (Tables S12 and S13). Furthermore, the results were consistent when incident CVD cases occurring within the first two years of follow‐up were excluded (Table S14).

## 4 Discussion

In this prospective cohort study of Chinese adults, data from three waves of the Kailuan Study were analyzed to assess dual trajectory patterns of seven CA indices combined with FPG and their associations with CVD. It was found that an elevated FPG level frequently coincides with higher adiposity indices. A higher FPG level was significantly associated with a subsequent elevated CVD risk. Additionally, compared with the dual trajectory patterns of WC combined with FPG, the dual trajectory patterns of WHtR and BRI combined with FPG demonstrated superior predictive performance for CVD. However, no significant differences were found in C statistics and IDI comparisons.

A previous study [29] showed consistent changes in BMI and WC across genders, with a similar distribution of participants in each group. Similarly, the optimal number of clusters and trajectory shape parameters were identified in this study. Among the seven CA indices, the co‐evolutionary patterns of four indices (WC, WHtR, AVI, and BRI) combined with FPG were grouped into four distinct dual trajectory pattern groups. Meanwhile, the co‐evolutionary patterns of the remaining three indices (WHR, C‐index, and ABSI) combined with FPG were classified into three distinct dual trajectory pattern groups. This study extends previous research by confirming and expanding the identification of distinct CA index trajectory patterns combined with FPG and their association with CVD risk in the Chinese population. Several studies have examined the trajectories of different CA indices in the Chinese population, focusing on WC [14] and BRI [16] in the general adult population, and WHR trajectories in heart failure patients [15], with consistent findings that a specific trajectory is associated with the highest risk. Furthermore, a longitudinal investigation [17] using participant age as a timescale for the trajectories revealed three distinct FPG trajectories in normoglycemic individuals, which were associated with coronary heart disease. Earlier findings from the Kailuan Study also outlined five discrete FPG trajectories associated with subsequent myocardial infarction risk in non‐diabetic individuals [18]. This study identified sets of three or four distinct dual trajectory patterns associated with CVD risk, possibly due to the concurrent examination of various CA indices and FPG changes.

Furthermore, the findings align with previous studies from diverse populations that highlight the significance of CA and glucose metabolism in cardiovascular risk prediction. For instance, Xu et al. [30] demonstrated that visceral adiposity is a critical determinant of cardiovascular outcomes, emphasizing the predictive superiority of indices reflecting central fat accumulation over BMI. Additionally, Wang et al. [31] explored various adiposity measures and found that indices, such as WHtR, were more strongly associated with heart failure outcomes than WC, supporting the observation that WHtR combined with FPG exhibits superior predictive performance versus WC combined with FPG. These results extend previous findings by simultaneously tracking the joint trajectories of these indices, providing a dynamic understanding of their concurrent progression and their implications for CVD risk.

The present study introduced a novel perspective by employing group‐based multi‐trajectory modeling to define distinct trajectory groups based on two indicators, capturing their interrelationships and visualizing their joint developmental patterns over time. It was found that different patterns of different CA indices combined with FPG level were associated with varying CVD risks. Compared to the reference group with the low CA indices and low FPG levels, the group with the moderate/high CA indices & high/highest FPG levels exhibited a higher risk of CVD. These results reaffirmed that both CA [10, 15, 16] and hyperglycemia [18] were associated with an increased CVD risk, and considering both factors enhances CVD risk stratification.

Furthermore, the predictive capabilities of dual trajectory patterns involving various CA indices combined with FPG for CVD development were assessed. The findings indicated that the dual trajectory models of WHtR combined with FPG and BRI demonstrated statistically superior predictive performance for CVD compared with WC combined with FPG. However, the clinical applicability and widespread use of WC as a central adiposity measure should not be overlooked. While WHtR and BRI require only an additional height measurement compared with WC, their implementation in clinical practice is less common. Given WC's extensive validation in epidemiological studies and its ease of measurement, WC combined with FPG remains an important and practical tool for risk assessment, despite marginal differences in predictive capability [10, 32, 33, 34].

This study confirmed that high levels of blood glucose and corpulence independently increase the risk of CVD. However, the dual trajectory approach provides additional insights by capturing longitudinal patterns in FPG and central adiposity indices, rather than relying on baseline or single‐point measurements. This approach realizes the identification of individuals who persistently exhibit high‐risk trajectories, which may provide insights for long‐term interventions.

Participants who maintain high FPG levels over time face a heightened risk of CVD, independent of corpulence trajectories, indicating that both factors independently contribute to cardiovascular risk. This finding supports the importance of monitoring both central adiposity and FPG levels in clinical practice. The independence of the high glucose trajectory underscores the need to consider glucose control as a separate therapeutic target. In addition, the persistence of the high corpulence trajectory highlights the clinical significance of sustained central adiposity in increasing CVD risk. This finding suggests that individuals with stable high corpulence are at an elevated risk and should be prioritized for early intervention strategies aimed at reducing central adiposity.

In the highest FPG trajectory group, a trend toward a decrease in FPG level was found between September 2008 and November 2010. This trend likely reflects the impact of therapeutic interventions aimed at managing blood glucose levels. These interventions may have moderated the FPG trajectory, suggesting that early medical management could influence CVD risk trajectories for individuals with high FPG. Future studies should further investigate the role of therapeutic interventions in modifying these trajectories to assess their potential for risk reduction.

While it is well‐established that high FPG and high corpulence are individually associated with increased CVD risk, this study provided additional insights by examining the joint trajectories of these factors over time. The dual trajectory approach allowed for the identification of distinct patterns of FPG and central adiposity development, demonstrating that participants with persistently high trajectories in both indices have a significantly higher risk of CVD compared to those with only one elevated trajectory. This suggests that individuals following a dual high‐risk trajectory may benefit from more targeted, multifaceted interventions concentrated on both glucose control and weight management. Furthermore, the joint trajectory analysis highlighted the importance of early intervention for individuals with moderate or increasing FPG and corpulence trajectories, who may still be at elevated risk even if they do not fall into the highest risk categories individually. For public health, these findings suggest the need for integrated strategies that address both blood glucose and central adiposity over time, rather than concentrating solely on a single risk factor at baseline.

## 5 Strengths and Limitations

The study pioneered the assessment of the dual trajectory patterns of various CA indices combined with FBG, providing a novel exploration of their joint impact on CVD. The dual trajectory modeling approach optimizes the CVD risk assessment by considering the dynamic interplay between the two risk factors rather than relying solely on cross‐sectional data for a single indicator. Furthermore, this study presented a comprehensive comparison of the predictive performance of various CA indices in CVD risk assessment, providing a basis for their clinical utility in evaluating CVD risk. The analysis is based on a large study sample with an extended follow‐up period and high‐quality longitudinal data from a prospectively designed, real‐world cohort.

However, the study has some limitations. Firstly, the analysis was based primarily on data from the Han Chinese population in northern China, which might limit the generalizability to the entire country or other ethnic groups. Secondly, visceral adipose tissue was not assessed using advanced methods, such as computed tomography or magnetic resonance imaging; instead, alternative, widely available, and cost‐effective indicators were used, providing practicality and simplicity in evaluating CA. Additionally, the influence of dietary patterns or physical activity trends over time was not analyzed, which might impact the observed trajectories. Future studies should incorporate these factors for a more comprehensive understanding. Lastly, the male skewness in the participant sample might limit the extrapolation of the findings. Further evaluation of the reproducibility of these findings in other populations is needed.

## 6 Implications for Clinical Practice

This study underscores the importance of tracking joint trajectories of CA indices and FPG for the prediction of CVD risk. This aligns with the recently published 2024 Chinese Guidelines for Obesity Diagnosis and Treatment, emphasizing that obesity is mainly accompanied by hypertension, dyslipidemia, and insulin resistance, which are key contributors to CVD risk. Clinical practice should prioritize long‐term monitoring and management of these indicators to effectively reduce CVD risk, rather than relying solely on single‐point measurements or individual risk factors.

## 7 Future Research Directions

Future research should investigate whether identifying concurrent increases in CA indices and FPG in clinical practice could assist clinicians in diagnosing conditions earlier or targeting interventions to reduce CVD risk. Additionally, it is essential to investigate whether modeling dual trajectories enhances risk prediction versus analyzing individual trajectories separately. Further exploration of genetic, lifestyle, and metabolic factors influencing these trajectories could provide valuable insights for more personalized preventive strategies.

## 8 Conclusions

In conclusion, sets of dual trajectory patterns involving various CA indices combined with FPG were identified, and these patterns were strongly associated with CVD development. The dual trajectories of WHtR and BRI combined with FPG demonstrated slightly superior predictive performance for CVD compared to WC combined with FPG. However, given the clinical practicality and widespread use of WC, it remains a valuable tool for risk stratification. These findings suggest that while alternative indices may provide slight improvements in predictive performance, WC‐based risk assessment continues to be a widely applicable and practical approach in clinical settings.

## Author Contributions

X.D. and Y.T. performed the statistical analysis. G.Z., X.D., H.Z., R. Z., J.M.N., T.B., S.W., and L.L.Y. interpreted the data. G.Z., X.D., and D.W. interpreted the findings and drafted the manuscript. G.Z., X.D., and L.L.Y. designed and supervised the study. All authors read the manuscript, edited it for intellectual content, and gave final approval for this version to be published. G.Z., S.W., and L.L.Y. are the guarantors of this study and had full access to all the data in the study. They take responsibility for the integrity of the data and the accuracy of the data analysis.

## Conflicts of Interest

The authors declare no conflicts of interest.

## Supporting information


**Data S1.** Supporting Information.

**Data S2.** Supporting Information.

## Data Availability

The data that support the findings of this study are available in Supporting Information S2.
