# Supplementary material for: Predictive Capability of Dual Trajectories of Central Adiposity Indices Combined With Glucose for Cardiovascular Diseases
Source: J Diabetes. 2025 Apr 25;17(4):e70081. doi: 10.1111/1753-0407.70081 (PMC12022674; doi:10.1111/1753-0407.70081)
Supplement: Supplementary file 1 — Data S1. Supporting Information. [file JDB-17-e70081-s001.docx]

Supplementary Table S1. Calculation of different CA Indices

| Central adiposity indices | Formula of calculation |
| --- | --- |
| WC | - |
| WHtR | $\frac{{WC}_{cm}}{{Ht}_{cm}}$ |
| AVI | $\frac{{{2\times(WC}_{cm})}^{2}+0.7\times({{WC}_{cm}-{HC}_{cm})}^{2}}{1000}$ |
| BRI | $364.2-365.5\times{[1-({\frac{\frac{{WC}_{m}}{2\pi}}{0.5\times{Ht}_{m}})}^{2}]}^{1/2}$ |
| WHR | $\frac{{WC}_{cm}}{{Ht}_{cm}}$ |
| C-index | $\frac{{WC}_{m}}{{0.109\times(\frac{{WC}_{kg}}{{Ht}_{m}})}^{1/2}}$ |
| ABSI | $\frac{{WC}_{m}}{{{{(Ht}_{m})}^{1/2}\times(\frac{{Wt}_{kg}}{{{Ht}_{m}}^{2}})}^{2/3}}$ |

This table describes the calculation of each of the central adiposity index.

Abbreviations: ABSI, a new body shape index; AVI, abdominal volume index; BRI, body roundness index; CA, Central adiposity; C-index, conicity index; WC, waist circumference; WHR, waist-to-hip ratio; WHtR, waist-to-height ratio; Ht: Height (m); HC: Hip Circumference (cm); Wt: Weight (kg).

Supplementary Table S2. Parameters of Model-Adequacy Criteria of the Dual trajectory Model

| Variables | Number of groups | Order of trajectory | Proportion of individuals in groups (%) | BIC | 2ΔBIC | AIC | Average posterior probability of assignment |
| --- | --- | --- | --- | --- | --- | --- | --- |
| WC & FPG | 2 | 1/2 | 56.3/43.7 | -617678.5 | NA | -617622.6 | 0.91/0.89 |
|  | 2 | 2/1 |  |  |  |  |  |
|  | 3 | 2/2/1 | 49.3/45.4/5.3 | -590062.5 | 27615.9 | -589980.9 | 0.90/0.89/0.97 |
|  | 3 | 1/2/1 |  |  |  |  |  |
|  | **4** | **1/1/1/1/** | **25.0/52.4/17.4/5.2** | **-586782.3** | **3280.2** | **-586687.8** | **0.86/0.85/0.85/0.97** |
|  | **4** | **2/1/1/1** |  |  |  |  |  |
|  | 5 | 1/1/1/1/2 | 24.2/50.9/16.8/5.8/2.3 | -581152.8 | 5629.5 | -581177.0 | 0.86/0.85/0.84/0.92/0.97 |
|  | 5 | 1/1/1/1/1 |  |  |  |  |  |
| WHtR & FPG | 2 | 1/1 | 94.2/5.8 | 10913.5 | NA | 10960.8 | 0.99/0.97 |
|  | 2 | 1/1 |  |  |  |  |  |
|  | 3 | 2/1/2 | 54.6/40.0/5.4 | 21138.3 | 10224.7 | 21219.9 | 0.90/0.88/0.97 |
|  | 3 | 1/2/1 |  |  |  |  |  |
|  | **4** | **1/1/1/2** | **27.2/53.7/13.9/5.2** | **24115.6** | **2977.3** | **24210.1** | **0.85/0.85/0.84/0.97** |
|  | **4** | **1/1/1/1** |  |  |  |  |  |
|  | 5 | 1/1/1/1/2 | 26.6/13.0/52.2/5.9/2.3 | 29777.6 | 5662.0 | 29893.6 | 0.84/0.83/0.84/0.92/0.97 |
|  | 5 | 1/1/1/1/1 |  |  |  |  |  |
| AVI & FPG | 2 | 1/1 | 94.2/5.8 | -476257.5 | NA | -476210.2 | 0.99/0.98 |
|  | 2 | 1/1 |  |  |  |  |  |
|  | 3 | 1/2/1 | 59.4/35.3/5.3 | -464605.8 | 11651.6 | -464528.5 | 0.92/0.88/0.97 |
|  | 3 | 1/2/1 |  |  |  |  |  |
|  | **4** | **1/1/1/1** | **34.0/48.6/12.3/5.2** | **-461449.8** | **3156.1** | **-461359.5** | **0.86/0.84/0.86/0.86** |
|  | **4** | **1/1/1/1** |  |  |  |  |  |
|  | 5 | 1/1/1/1/2 | 33.4/47.0/11.7/5.7/2.2 | -455916.0 | 5533.8 | -455800.0 | 0.86/0.84/0.85/0.92/0.97 |
|  | 5 | 1/1/1/1/1 |  |  |  |  |  |
| BRI & FPG | 2 | 1/2 | 94.2/5.8 | -345667.0 | NA | -345615.5 | 0.99/0.98 |
|  | 2 | 1/1 |  |  |  |  |  |
|  | 3 | 1/1/1 | 65.0/29.7/5.3 | -335380.0 | 10287.0 | -335302.7 | 0.93/0.87/0.97 |
|  | 3 | 1/2/1 |  |  |  |  |  |
|  | **4** | **1/1/1/2** | **39.1/46.8/8.9/5.2** | **-332579.2** | **2800.8** | **-332484.7** | **0.86/0.83/0.85/0.97** |
|  | **4** | **1/1/1/1** |  |  |  |  |  |
|  | 5 | 1/1/2/1/2 | 38.7/45.0/8.2/5.8/2.3 | -326981.6 | 5597.6 | -326869.9 | 0.85/0.83/0.85/0.92/0.97 |
|  | 5 | 1/1/1/2/2 |  |  |  |  |  |
| WHR & FPG | 2 | 1/2 | 94.4/5.6 | -9701.2 | NA | -9649.6 | 0.99/0.97 |
|  | 2 | 1/1 |  |  |  |  |  |
|  | **3** | **1/1/2** | **22.3/72.2/5.5** | **-7337.2** | **2364.0** | **-7259.9** | **0.76/0.88/0.97** |
|  | **3** | **2/1/1** |  |  |  |  |  |
|  | 4 | 1/1/1/1 | 18.2/72.9/6.5/2.4 | -1017.4 | 6319.8 | -927.2 | 0.76/0.90/0.93/0.97 |
|  | 4 | 1/1/1/1 |  |  |  |  |  |
| C-index & FPG | 2 | 1/1 | 94.4/5.6 | -610468.3 | NA | -610403.9 | 0.99/0.97 |
|  | 2 | 1/1 |  |  |  |  |  |
|  | **3** | **1/1/1** | **67.2/27.3/5.5** | **-608294.1** | **2174.2** | **-608225.4** | **0.86/0.76/0.97** |
|  | **3** | **1/1/1** |  |  |  |  |  |
|  | 4 | 1/1/1/1 | 67.4/6.6/23.6/2.4 | -601949.2 | 6344.9 | -601859.0 | 0.87/0.92/0.75/0.97 |
|  | 4 | 1/1/1/1 |  |  |  |  |  |
| ABSI & FPG | 2 | 1/1 | 94.4/5.6 | 25856.1 | NA | 25903.4 | 0.99/0.97 |
|  | 2 | 1/1 |  |  |  |  |  |
|  | **3** | **1/1/1** | **79.3/15.1/5.6** | **29721.4** | **3865.3** | **29790.2** | **0.94/0.81/0.97** |
|  | **3** | **1/1/1** |  |  |  |  |  |
|  | 4 | 1/1/1/1 | 76.7/14.4/6.5/2.4 | 36051.0 | 6329.5 | 36141.2 | 0.94/0.81/0.92/0.98 |
|  | 4 | 1/1/1/1 |  |  |  |  |  |

Abbreviations: ABSI, A new body shape index; AIC, Akaike information criterion; AVI, Abdominal volume index; BRI, Body roundness index; BIC, Bayesian information criterion; C-index, Conicity index; FPG, Fasting plasma glucose; WC, Waist circumference; WHR, Waist-to-hip ratio; WHtR, Waist-to-height ratio.

Supplementary Table S3. Baseline Characteristics of Participants in Multiple Imputation Missing Covariates Before and After

| **Characteristics** | **Multiple imputation before**  **(n=39 772)** | **Multiple imputation after**  **(n=39 772)** |
| --- | --- | --- |
| Age, mean (SD), y | 52.6 (11.6) | 52.6 (11.6) |
| Sex |  |  |
| Female | 8301 (20.9) | 8301 (20.9) |
| Male | 31 471 (79.1) | 31 471 (79.1) |
| Education background |  |  |
| Illiterate/elementary | 2808 (7.0) | 2808 (7.0) |
| Middle school | 27 433 (69.0) | 27 433 (69.0) |
| Vocational /high school above | 9531 (24.0) | 9531 (24.0) |
| Marital status |  |  |
| Never married/widowed/separated | 473 (1.2) | 473 (1.2) |
| Married or remarriage | 39 299 (98.8) | 39 299 (98.8) |
| Current smoking |  |  |
| No | 25 755 (64.8) | 25 768 (64.8) |
| Yes | 13 994 (35.2) | 14 004 (35.2) |
| Missing^*^ | 23 | NA |
| Current drinking |  |  |
| No | 25 574 (64.3) | 25 589 (64.3) |
| Yes | 14 174 (35.7) | 14 183 (35.7) |
| Missing^*^ | 24 | NA |
| Physical activities |  |  |
| No | 12 412 (31.2) | 12 420 (31.2) |
| Occasionally | 21 999 (55.4) | 22 014 (55.4) |
| Frequently | 5335 (13.4) | 5338 (13.4) |
| Missing^*^ | 26 | NA |
| Salt intake habits |  |  |
| high | 4194 (10.6) | 4198 (10.6) |
| Medium | 28 613 (72.0) | 28 635 (72.0) |
| Low | 6937 (17.4) | 6939 (17.4) |
| Missing^*^ | 28 | NA |
| BMI, mean (SD),·kg/m^2^ | 25.1 (3.2) | 25.1 (3.2) |
| SBP, mean (SD), mm Hg | 130.5 (18.1) | 130.6 (18.2) |
| SBP, missing | 371 (0.9) | NA |
| DBP, mean (SD), mm Hg | 84.4 (10.3) | 84.4 (10.3) |
| DBP, missing | 366 (0.9) | NA |
| LDL-C, mean (SD), mmol/L | 2.6 (0.8) | 2.6 (0.8) |
| LDL-C, missing | 404 (1.0) | NA |
| HDL-C, mean (SD), mmol/L | 1.5 (0.4) | 1.5 (0.4) |
| HDL-C, missing | 286 (0.7) | NA |
| Hs-CRP, median (IQR), mg/L | 1.0 (0.4-2.4) | 1.02 (0.5-2.5) |
| Hs-CRP, missing | 262 (0.7) | NA |
| eGFR, median (IQR), ml/min/1.73 m^3^ | 90.9 (75.0-103.4) | 90.9 (75.0-103.4) |
| eGFR, missing | 353 (0.9) | NA |
| Hypertension | 18 962 (47.7) | 18 962 (47.7) |
| Diabetes mellitus | 4242 (10.7) | 4242 (10.7) |
| Medication use |  |  |
| Antihypertensive | 4035 (10.1) | 4035 (10.1) |
| Antidiabetic | 1152 (2.9) | 1152 (2.9) |
| Lipid-lowering | 326 (0.8) | 326 (0.8) |

Abbreviations: BMI, body mass index; DBP, diastolic blood pressure; eGFR, estimated glomerular filtration rate; HDL-C, high-density lipoprotein cholesterol; Hs-CRP, high-sensitivity C-reactive protein; LDL-C, low-density lipoprotein cholesterol; SBP, systolic blood pressure. ^*^ Missing < 0.1%

Data are presented as number (%) unless otherwise specified.

Supplementary Table S4. Baseline Characteristics of the Study Participants According to Dual trajectory Patterns of Waist Circumference and Fasting Plasma Glucose

| **Characteristics** | **Low WC & low FPG group**  **(n = 9473)** | **Moderate WC & moderate FPG group**  **(n = 21 756)** | **Highest WC & high FPG group**  **(n = 6465)** | **High WC & highest FPG group**  **(n = 2078)** |
| --- | --- | --- | --- | --- |
| Age, mean (SD), y | 49.1 (11.7) | 53.0 (11.4) | 54.8 (11.7) | 57.9 (9.5) |
| Male | 5917 (62.5) | 18 084 (83.1) | 5770 (89.3) | 1700 (81.8) |
| Education background |  |  |  |  |
| Illiterate/elementary | 521 (5.5) | 1532 (7.0) | 561 (8.7) | 194 (9.3) |
| Middle school | 5970 (63.0) | 15 273 (70.2) | 4610 (71.3) | 1580 (76.0) |
| Vocational /high school above | 2982 (31.5) | 4951 (22.8) | 1294 (20.0) | 304 (14.6) |
| Married or remarriage | 9292 (98.1) | 21 532 (99.0) | 6414 (99.2) | 2061 (99.2) |
| Current smoking | 2848 (30.1) | 7924 (36.4) | 2555 (39.5) | 677 (32.6) |
| Current drinking | 2739 (28.9) | 8191 (37.6) | 2638 (40.8) | 615 (29.6) |
| Physical activities |  |  |  |  |
| No | 3164 (33.4) | 6599 (30.3) | 2084 (32.2) | 573 (27.6) |
| Occasionally | 5171 (54.6) | 12 180 (56.0) | 3481 (53.8) | 1182 (56.9) |
| Frequently | 1138 (12.0) | 2977 (13.7) | 900 (13.9) | 323 (15.5) |
| Salt intake habits |  |  |  |  |
| High | 905 (9.5) | 2327 (10.7) | 750 (11.6) | 216 (10.4) |
| Medium | 6782 (71.6) | 15 634 (71.9) | 4722 (73.0) | 1497 (72.0) |
| Low | 1786 (18.9) | 3795 (17.4) | 993 (15.4) | 365 (17.6) |
| BMI, mean (SD), kg/m2 | 22.3 (2.2) | 25.2 (2.5) | 28.5 (2.7) | 26.0 (3.1) |
| SBP, mean (SD), mm Hg | 122.9 (16.9) | 131.1 (17.5) | 137.3 (17.7) | 139.6 (19.3) |
| DBP, mean (SD), mm Hg | 80.2 (9.8) | 84.9 (9.9) | 88.4 (10.3) | 86.5 (10.4) |
| LDL-C, mean (SD), mmol/L | 2.5 (0.7) | 2.6 (0.8) | 2.7 (0.8) | 2.7 (0.8) |
| HDL-C, mean (SD), mmol/L | 1.6 (0.4) | 1.5 (0.4) | 1.4 (0.4) | 1.5 (0.4) |
| Hs-CRP, median (IQR), mg/L | 0.7 (0.3-1.6) | 1.0 (0.5-2.5) | 1.6 (0.7-3.3) | 1.4 (0.7-3.2) |
| eGFR, median (IQR), ml/min/1.73 m^3^ | 94.9 (77.5-106.9) | 89.9 (74.3-102.5) | 90.0 (75.6-101.9) | 87.8 (72.1-99.8) |
| Hypertension | 2746 (29.0) | 10550 (48.5) | 4292 (66.4) | 1374 (66.1) |
| Diabetes mellitus | 220 (2.3) | 1229 (5.6) | 830 (12.8) | 1963 (94.5) |
| Medication use |  |  |  |  |
| Antihypertensive | 406 (4.3) | 2057 (9.5) | 1183 (18.3) | 389 (18.7) |
| Antidiabetic | 36 (0.4) | 223 (1.0) | 166 (2.6) | 727 (35.0) |
| Lipid-lowering | 26 (0.3) | 159 (0.7) | 91 (1.4) | 50 (2.4) |

Abbreviations: BMI, body mass index; DBP, diastolic blood pressure; eGFR, estimated glomerular filtration rate; HDL-C, high-density lipoprotein cholesterol; Hs-CRP, high-sensitivity C-reactive protein; LDL-C, low-density lipoprotein cholesterol; SBP, systolic blood pressure.

Data are presented as number (%) unless otherwise specified.

Supplementary Table S5. Baseline Characteristics of the Study Participants According to Dual trajectory Patterns of Waist-Height Ratio and Fasting Plasma Glucose

| **Characteristics** | **Low WHtR & low FPG group**  **(n = 10 323)** | **Moderate WHtR & moderate FPG group**  **(n = 22 302)** | **Highest WHtR & high FPG group (n = 5065)** | **High WHtR & highest FPG group**  **(n = 2082)** |
| --- | --- | --- | --- | --- |
| Age, mean (SD), y | 48.4 (11.7) | 53.05 ± 11.20 | 57.24 ± 11.29 | 57.93 ± 9.62 |
| Male | 7856 (76.1) | 18 174 (81.49) | 3725 (73.54) | 1716 (82.42) |
| Education background |  |  |  |  |
| Illiterate/elementary | 519 (5.0) | 1528 (6.85) | 566 (11.17) | 195 (9.37) |
| Middle school | 6416 (62.2) | 15 722 (70.50) | 3715 (73.35) | 1580 (75.89) |
| Vocational /high school above | 3388 (32.8) | 5052 (22.65) | 784 (15.48) | 307 (14.75) |
| Married or remarriage | 10132 (98.1) | 220 84 (99.02) | 5019 (99.09) | 2064 (99.14) |
| Current smoking | 3798 (36.8) | 7968 (35.73) | 1561 (30.82) | 677 (32.52) |
| Current drinking | 3659 (35.4) | 8301 (37.22) | 1602 (31.63) | 621 (29.83) |
| Physical activities |  |  |  |  |
| No | 3481 (33.7) | 6764 (30.33) | 1608 (31.75) | 567 (27.23) |
| Occasionally | 5601 (54.3) | 12 520 (56.14) | 2699 (53.29) | 1194 (57.35) |
| Frequently | 1241 (12.0) | 3018 (13.53) | 758 (14.97) | 321 (15.42) |
| Salt intake habits |  |  |  |  |
| High | 1026 (9.9) | 2367 (10.61) | 590 (11.65) | 215 (10.33) |
| Medium | 7290 (70.6) | 16 130 (72.33) | 3713 (73.31) | 1502 (72.14) |
| Low | 2007 (19.5) | 3805 (17.06) | 762 (15.04) | 365 (17.53) |
| BMI, mean (SD), kg/m2 | 22.3 (2.2) | 25.44 ± 2.49 | 28.78 ± 2.85 | 26.02 ± 3.06 |
| SBP, mean (SD), mm Hg | 123.4 (16.4) | 131.35 ± 17.63 | 138.33 ± 18.35 | 139.56 ± 19.28 |
| DBP, mean (SD), mm Hg | 81.0 (9.7) | 85.08 ± 10.16 | 87.87 ± 10.32 | 86.50 ± 10.41 |
| LDL-C, mean (SD), mmol/L | 2.5 (0.7) | 2.63 ± 0.76 | 2.66 ± 0.87 | 2.73 ± 0.83 |
| HDL-C, mean (SD), mmol/L | 1.6 (0.4) | 1.50 ± 0.40 | 1.44 ± 0.38 | 1.46 ± 0.40 |
| Hs-CRP, median (IQR), mg/L | 0.7 (0.3-1.5) | 1.10 (0.50, 2.56) | 1.70 (0.81, 3.58) | 1.40 (0.70, 3.29) |
| eGFR, median (IQR), ml/min/1.73 m^3^ | 95.3 (77.8-107.4) | 89.98 (74.48, 102.54) | 88.64 (74.25, 100.12) | 87.44 (72.07, 99.57) |
| Hypertension | 3079 (29.8) | 11 066 (49.62) | 3443 (67.98) | 1374 (65.99) |
| Diabetes mellitus | 242 (2.3) | 1265 (5.67) | 773 (15.26) | 1962 (94.24) |
| Medication use |  |  |  |  |
| Antihypertensive | 409 (4.0) | 2208 (9.90) | 1031 (20.36) | 387 (18.59) |
| Antidiabetic | 35 (0.3) | 224 (1.00) | 169 (3.34) | 724 (34.77) |
| Lipid-lowering | 27 (0.3) | 170 (0.76) | 79 (1.56) | 50 (2.40) |

Abbreviations: BMI, body mass index; DBP, diastolic blood pressure; eGFR, estimated glomerular filtration rate; HDL-C, high-density lipoprotein cholesterol; Hs-CRP, high-sensitivity C-reactive protein; LDL-C, low-density lipoprotein cholesterol; SBP, systolic blood pressure.

Data are presented as number (%) unless otherwise specified.

Supplementary Table S6. Baseline Characteristics of The Study Participants According to Dual trajectory Patterns of Abdominal Volume Index and Fasting Plasma Glucose

| **Characteristics** | **Low AVI & low FPG group**  **(n = 13 199)** | **Moderate AVI & moderate FPG group**  **(n = 20 005)** | **Highest AVI & high FPG group**  **(n = 4509)** | **High AVI & highest FPG group**  **(n = 2059)** |
| --- | --- | --- | --- | --- |
| Age, mean (SD), y | 50.0 (11.8) | 53.3 (11.3) | 55.1 (11.8) | 57.9 (9.5) |
| Male | 8791 (66.6) | 16 947 (84.7) | 4048 (89.8) | 1685 (81.8) |
| Education background |  |  |  |  |
| Illiterate/elementary | 736 (5.6) | 1473 (7.4) | 404 (9.0) | 195 (9.5) |
| Middle school | 8558 (64.8) | 14 068 (70.3) | 3243 (71.9) | 1564 (76.0) |
| Vocational /high school above | 3905 (29.6) | 4464 (22.3) | 862 (19.1) | 300 (14.6) |
| Married or remarriage | 12 981 (98.3) | 19 806 (99.0) | 4470 (99.1) | 2042 (99.2) |
| Current smoking | 4142 (31.4) | 7422 (37.1) | 1771 (39.3) | 669 (32.5) |
| Current drinking | 4017 (30.4) | 7753 (38.8) | 1802 (40.0) | 611 (29.7) |
| Physical activities |  |  |  |  |
| No | 4317 (32.7) | 6047 (30.2) | 1488 (33.0) | 568 (27.6) |
| Occasionally | 7244 (54.9) | 11 213 (56.1) | 2383 (52.8) | 1174 (57.0) |
| Frequently | 1638 (12.4) | 2745 (13.7) | 638 (14.2) | 317 (15.4) |
| Salt intake habits |  |  |  |  |
| High | 1264 (9.6) | 2189 (10.9) | 531 (11.8) | 214 (10.4) |
| Medium | 9442 (71.5) | 14 420 (72.1) | 3291 (73.0) | 1482 (72.0) |
| Low | 2493 (18.9) | 3396 (17.0) | 687 (15.2) | 363 (17.6) |
| BMI, mean (SD), kg/m2 | 22.7 (2.3) | 25.7 (2.5) | 28.9 (2.7) | 26.0 (3.2) |
| SBP, mean (SD), mm Hg | 124.4 (17.2) | 132.1 (17.6) | 138.1 (17.7) | 139.5 (19.3) |
| DBP, mean (SD), mm Hg | 81.0 (9.9) | 85.5 (10.0) | 88.8 (10.3) | 86.5 (10.4) |
| LDL-C, mean (SD), mmol/L | 2.5 (0.7) | 2.6 (0.8) | 2.7 (0.8) | 2.7 (0.8) |
| HDL-C, mean (SD), mmol/L | 1.6 (0.4) | 1.5 (0.4) | 1.4 (0.4) | 1.5 (0.4) |
| Hs-CRP, median (IQR), mg/L | 0.8 (0.3-1.7) | 1.1 (0.5-2.6) | 1.7 (0.8-3.5) | 1.4 (0.7-3.2) |
| eGFR, median (IQR), ml/min/1.73 m^3^ | 93.5 (76.1-106.0) | 90.0 (74.7-102.3) | 89.9 (75.1-101.7) | 87.8 (72.2-99.9) |
| Hypertension | 4236 (32.1) | 10 281 (51.4) | 3085 (68.4) | 1360 (66.0) |
| Diabetes mellitus | 348 (2.6) | 1328 (6.6) | 623 (13.8) | 1943 (94.4) |
| Medication use |  |  |  |  |
| Antihypertensive | 642 (4.9) | 2138 (10.7) | 869 (19.3) | 386 (18.7) |
| Antidiabetic | 59 (0.4) | 244 (1.2) | 126 (2.8) | 723 (35.1) |
| Lipid-lowering | 50 (0.4) | 165 (0.8) | 61 (1.3) | 50 (2.4) |

Abbreviations: BMI, body mass index; DBP, diastolic blood pressure; eGFR, estimated glomerular filtration rate; HDL-C, high-density lipoprotein cholesterol; Hs-CRP, high-sensitivity C-reactive protein; LDL-C, low-density lipoprotein cholesterol; SBP, systolic blood pressure.

Data are presented as number (%) unless otherwise specified.

Supplementary Table S7. Baseline Characteristics of the Study Participants According to Dual trajectory Patterns of Body Roundness Index and Fasting Plasma Glucose

| **Characteristics** | **Low BRI & low FPG group**  **(n=15 357)** | **Moderate BRI & moderate FPG group**  **(n=19 091)** | **Highest BRI & high FPG group**  **(n=3250)** | **High BRI & highest FPG group**  **(n=2074)** |
| --- | --- | --- | --- | --- |
| Age, mean (SD), y | 49.5 (11.6) | 53.7 (11.2) | 57.8 (11.3) | 57.9 (9.6) |
| Male | 11 963 (77.9) | 15 490 (81.1) | 2305 (70.9) | 1713 (82.6) |
| Education background |  |  |  |  |
| Illiterate/elementary | 813 (5.3) | 1396 (7.3) | 402 (12.4) | 197 (9.5) |
| Middle school | 9921 (64.6) | 13 563 (71.0) | 2377 (73.1) | 1572 (75.8) |
| Vocational /high school above | 4623 (30.1) | 4132 (21.6) | 471 (14.5) | 305 (14.7) |
| Married or remarriage | 15 108 (98.4) | 18 914 (99.1) | 3220 (99.1) | 2057 (99.2) |
| Current smoking | 5698 (37.1) | 6689 (35.0) | 942 (29.0) | 675 (32.5) |
| Current drinking | 5583 (36.3) | 7018 (36.8) | 965 (29.7) | 617 (29.7) |
| Physical activities |  |  |  |  |
| No | 4970 (32.4) | 5844 (30.6) | 1037 (31.9) | 569 (27.5) |
| Occasionally | 8469 (55.1) | 10 624 (55.7) | 1734 (53.4) | 1187 (57.2) |
| Frequently | 1918 (12.5) | 2623 (13.7) | 479 (14.7) | 318 (15.3) |
| Salt intake habits |  |  |  |  |
| High | 1548 (10.1) | 2040 (10.7) | 396 (12.2) | 214 (10.3) |
| Medium | 10 904 (71.0) | 13 870 (72.6) | 2363 (72.7) | 1498 (72.2) |
| Low | 2905 (18.9) | 3181 (16.7) | 491 (15.1) | 362 (17.5) |
| BMI, mean (SD), kg/m^2^ | 22.9 (2.3) | 26.0 (2.5) | 29.3 (2.9) | 26.0 (3.0) |
| SBP, mean (SD), mm Hg | 125.0 (16.9) | 132.7 (17.7) | 139.1 (18.4) | 139.6 (19.3) |
| DBP, mean (SD), mm Hg | 81.9 (9.9) | 85.7 (10.1) | 88.0 (10.5) | 86.5 (10.4) |
| LDL-C, mean (SD), mmol/L | 2.5 (0.7) | 2.6 (0.8) | 2.7 (0.9) | 2.7 (0.8) |
| HDL-C, mean (SD), mmol/L | 1.6 (0.4) | 1.5 (0.4) | 1.4 (0.4) | 1.5 (0.4) |
| Hs-CRP, median (IQR), mg/L | 0.8 (0.3-1.7) | 1.2 (0.6-2.7) | 1.8 (0.9-3.8) | 1.4 (0.7-3.2) |
| eGFR, median (IQR), ml/min/1.73 m^3^ | 93.7 (76.5-106.1) | 89.84 (74.6- 102.2) | 88.3 (73.9-99.9) | 87.4 (72.0, 99.6) |
| Hypertension | 5181 (33.7) | 10 144 (53.1) | 2268 (69.8) | 1369 (66.0) |
| Diabetes mellitus | 414 (2.7) | 1324 (6.9) | 550 (16.9) | 1954 (94.2) |
| Medication use |  |  |  |  |
| Antihypertensive | 727 (4.7) | 2200 (11.5) | 721 (22.2) | 387 (18.7) |
| Antidiabetic | 64 (0.4) | 244 (1.3) | 123 (3.8) | 721 (34.8) |
| Lipid-lowering | 52 (0.3) | 173 (0.9) | 52 (1.6) | 49 (2.4) |

Abbreviations: BMI, body mass index; DBP, diastolic blood pressure; eGFR, estimated glomerular filtration rate; HDL-C, high-density lipoprotein cholesterol; Hs-CRP, high-sensitivity C-reactive protein; LDL-C, low-density lipoprotein cholesterol; SBP, systolic blood pressure.

Data are presented as number (%) unless otherwise specified.

Supplementary Table S8. Baseline Characteristics of the Study Participants According to Dual trajectory Patterns of Waist-Hip Ratio and Fasting Plasma Glucose

| **Characteristics** | **Low WHR & low FPG group**  **(n = 7049)** | **High WHR & moderate FPG group**  **(n = 30 554)** | **High WHR & high FPG group**  **(n = 2169)** |
| --- | --- | --- | --- |
| Age, mean (SD), y | 49.9 (12.2) | 52.9 (11.5) | 57.9 (9.7) |
| Male | 3829 (54.3) | 25 867 (84.7) | 1775 (81.8) |
| Education background |  |  |  |
| Illiterate/elementary | 367 (5.2) | 2233 (7.3) | 208 (9.6) |
| Middle school | 4295 (60.9) | 21 495 (70.3) | 1643 (75.7) |
| Vocational /high school above | 2387 (33.9) | 6826 (22.3) | 318 (14.7) |
| Married or remarriage | 6936 (98.4) | 30 213 (98.9) | 2150 (99.1) |
| Current smoking | 1761 (25.0) | 11 539 (37.8) | 704 (32.5) |
| Current drinking | 1713 (24.3) | 11 835 (38.7) | 635 (29.3) |
| Physical activities |  |  |  |
| No | 2318 (32.9) | 9505 (31.1) | 597 (27.5) |
| Occasionally | 3710 (52.6) | 17 067 (55.9) | 1237 (57.0) |
| Frequently | 1021 (14.5) | 3982 (13.0) | 335 (15.4) |
| Salt intake habits |  |  |  |
| High | 631 (9.0) | 3340 (10.9) | 227 (10.5) |
| Medium | 5219 (74.0) | 21 856 (71.5) | 1560 (71.9) |
| Low | 1199 (17.0) | 5358 (17.5) | 382 (17.6) |
| BMI, mean (SD), kg/m^2^ | 23.4 (3.0) | 25.4 (3.1) | 26.1 (3.1) |
| SBP, mean (SD), mm Hg | 122.9 (17.3) | 131.7 (17.8) | 139.5 (19.3) |
| DBP, mean (SD), mm Hg | 80.1 (9.9) | 85.3 (10.2) | 86.5 (10.4) |
| LDL-C, mean (SD), mmol/L | 2.4 (0.7) | 2.6 (0.7) | 2.7 (0.8) |
| HDL-C, mean (SD), mmol/L | 1.6 (0.4) | 1.5 (0.4) | 1.5 (0.4) |
| Hs-CRP, median (IQR), mg/L | 0.8 (0.4-1.8) | 1.1 (0.5-2.6) | 1.4 (0.7-3.2) |
| eGFR, median (IQR), ml/min/1.73 m^3^ | 94.8 (79.2-106.4) | 90.2 (74.5-102.9) | 87.7 (72.0-99.8) |
| Hypertension | 2196 (31.1) | 15 325 (50.2) | 1441 (66.4) |
| Diabetes mellitus | 81 (1.1) | 2120 (6.9) | 2041 (94.1) |
| Medication use |  |  |  |
| Antihypertensive | 438 (6.2) | 3186 (10.4) | 411 (18.9) |
| Antidiabetic | 16 (0.2) | 387 (1.3) | 749 (34.5) |
| Lipid-lowering | 35 (0.5) | 240 (0.8) | 51 (2.3) |

Abbreviations: BMI, body mass index; DBP, diastolic blood pressure; eGFR, estimated glomerular filtration rate; HDL-C, high-density lipoprotein cholesterol; Hs-CRP, high-sensitivity C-reactive protein; LDL-C, low-density lipoprotein cholesterol; SBP, systolic blood pressure.

Data are presented as number (%) unless otherwise specified.

Supplementary Table S9. Baseline Characteristics of The Study Participants According to Dual trajectory Patterns of Conicity Index and Fasting Plasma Glucose

| **Characteristics** | **Low C-index & low FPG group**  **(n = 28 531)** | **High C-index & moderate FPG group**  **(n = 9039)** | **Moderate C-index & High FPG group**  **(n = 2202)** |
| --- | --- | --- | --- |
| Age, mean (SD), y | 50.7 (11.4) | 57.6 ± 11.0 | 57.9 (9.6) |
| Male | 22 157 (77.7) | 7512 (83.1) | 1802 (81.8) |
| Education background |  |  |  |
| Illiterate/elementary | 1659 (5.8) | 933 (10.3) | 216 (9.81) |
| Middle school | 19 071 (66.8) | 6696 (74.1) | 1666 (75.7) |
| Vocational /high school above | 7801 (27.3) | 1410 (15.6) | 320 (14.5) |
| Married or remarriage | 28 158 (98.7) | 8958 (99.1) | 2183 (99.1) |
| Current smoking | 9936 (34.8) | 3354 (37.1) | 714 (32.4) |
| Current drinking | 10 244 (35.9) | 3288 (36.4) | 651 (29.6) |
| Physical activities |  |  |  |
| No | 9015 (31.6) | 2797 (30.9) | 608 (27.6) |
| Occasionally | 15 820 (55.4) | 4939 (54.7) | 1255 (57.0) |
| Frequently | 3696 (13.0) | 1303 (14.4) | 339 (15.4) |
| Salt intake habits |  |  |  |
| High | 2990 (10.5) | 980 (10.8) | 228 (10.4) |
| Medium | 20 398 (71.5) | 6649 (73.6) | 1588 (72.1) |
| Low | 5143 (18.0) | 1410 (15.6) | 386 (17.5) |
| BMI, mean (SD), kg/m^2^ | 24.9 (3.1) | 25.5 (3.3) | 26.1 (3.1) |
| SBP, mean (SD), mm Hg | 128.6 (17.6) | 134.7 (18.5) | 139.5 (19.2) |
| DBP, mean (SD), mm Hg | 83.8 (10.2) | 85.9 (10.3) | 86.5 (10.4) |
| LDL-C, mean (SD), mmol/L | 2.6 (0.7) | 2.6 (0.9) | 2.7 (0.8) |
| HDL-C, mean (SD), mmol/L | 1.5 (0.4) | 1.5 (0.4) | 1.5 (0.4) |
| Hs-CRP, median (IQR), mg/L | 1.0 (0.4-2.2) | 1.4 (0.6-3.1) | 1.4 (0.7-3.2) |
| eGFR, median (IQR), ml/min/1.73 m^3^ | 91.8 (75.0-104.8) | 89.4 (75.9-99.9) | 87.8 (72.1-99.8) |
| Hypertension | 12 373 (43.3) | 5129 (56.7) | 1460 (66.3) |
| Diabetes mellitus | 1149 (4.0) | 1021 (11.3) | 2072 (94.1) |
| Medication use |  |  |  |
| Antihypertensive | 2299 (8.1) | 1329 (14.7) | 407 (18.5) |
| Antidiabetic | 190 (0.7) | 203 (2.2) | 759 (34.5) |
| Lipid-lowering | 179 (0.6) | 96 (1.1) | 51 (2.3) |

Abbreviations: BMI, body mass index; DBP, diastolic blood pressure; eGFR, estimated glomerular filtration rate; HDL-C, high-density lipoprotein cholesterol; Hs-CRP, high-sensitivity C-reactive protein; LDL-C, low-density lipoprotein cholesterol; SBP, systolic blood pressure.

Data are presented as number (%) unless otherwise specified.

Supplementary Table S10. Baseline Characteristics of the Study Participants According to Dual trajectory Patterns of A New Body Shape Index and Fasting Plasma Glucose

| **Characteristics** | **Low ABSI & low FPG group**  **(n = 32 554)** | **High ABSI & low FPG group**  **(n = 5006)** | **Moderate ASBI & high FPG group**  **(n = 2212)** |
| --- | --- | --- | --- |
| Age, mean (SD), y | 51.3 (11.3) | 59.2 (11.6) | 58.0 (9.6) |
| Male | 26 447 (81.2) | 3212 (64.2) | 1812 (81.9) |
| Education background |  |  |  |
| Illiterate/elementary | 2027 (6.2) | 566 (11.3) | 215 (9.7) |
| Middle school | 22 026 (67.7) | 3732 (74.6) | 1675 (75.7) |
| Vocational /high school above | 8501 (26.1) | 708 (14.1) | 322 (14.6) |
| Married or remarriage | 32 150 (98.8) | 4955 (99.0) | 2194 (99.2) |
| Current smoking | 11 902 (36.6) | 1386 (27.7) | 716 (32.3) |
| Current drinking | 12 277 (37.7) | 1253 (25.0) | 653 (29.5) |
| Physical activities |  |  |  |
| No | 10 351 (31.8) | 1455 (29.1) | 614 (27.8) |
| Occasionally | 17 931 (55.1) | 2825 (56.4) | 1258 (56.9) |
| Frequently | 4272 (13.1) | 726 (14.5) | 340 (15.4) |
| Salt intake habits |  |  |  |
| High | 3544 (10.9) | 425 (8.5) | 229 (10.3) |
| Medium | 23 226 (71.3) | 3812 (76.1) | 1597 (72.2) |
| Low | 5784 (17.8) | 769 (15.4) | 386 (17.5) |
| BMI, mean (SD), kg/m^2^ | 25.2 (3.1) | 23.9 (3.1) | 26.1 (3.1) |
| SBP, mean (SD), mm Hg | 129.8 (17.8) | 131.8 (19.1) | 139.5 (19.2) |
| DBP, mean (SD), mm Hg | 84.5 (10.3) | 83.4 (10.1) | 86.5 (10.4) |
| LDL-C, mean (SD), mmol/L | 2.6 (0.7) | 2.5 (0.9) | 2.7 (0.8) |
| HDL-C, mean (SD), mmol/L | 1.5 (0.4) | 1.5 (0.4) | 1.5 (0.4) |
| Hs-CRP, median (IQR), mg/L | 1.0 (0.4-2.3) | 1.3 (0.6-3.2) | 1.4 (0.7-3.2) |
| eGFR, median (IQR), ml/min/1.73 m^3^ | 91.8 (75.3-104.4) | 88.0 (74.9-98.5) | 87.7 (72.0-99.6) |
| Hypertension | 15 115 (46.4) | 2379 (47.5) | 1468 (66.4) |
| Diabetes mellitus | 1853 (5.7) | 306 (6.1) | 2083 (94.2) |
| Medication use |  |  |  |
| Antihypertensive | 3012 (9.2) | 607 (12.1) | 416 (18.8) |
| Antidiabetic | 329 (1.0) | 59 (1.2) | 764 (34.5) |
| Lipid-lowering | 237 (0.7) | 37 (0.7) | 52 (2.3) |

Abbreviations: BMI, body mass index; DBP, diastolic blood pressure; eGFR, estimated glomerular filtration rate; HDL-C, high-density lipoprotein cholesterol; Hs-CRP, high-sensitivity C-reactive protein; LDL-C, low-density lipoprotein cholesterol; SBP, systolic blood pressure.

Data are presented as number (%) unless otherwise specified.

Supplementary Table S11. Adjusted Hazard Ratios (HRs) and 95% Confidence Intervals (95% CIs) of Cardiovascular Diseases According to Dual Trajectory Patterns of Various Central Adiposity Indices and Fasting Plasma Glucose

| **Dual trajectory groups** | **Case/N** | **Incidence rate (95% CI),**  **per 1000 person-years** | **Adjusted HR (95% CI)^a^** | **Adjusted HR (95% CI)^b^** | **Adjusted HR (95% CI)^c^** |
| --- | --- | --- | --- | --- | --- |
| WC & FPG |  |  |  |  |  |
| Low WC & low FPG group | 343/9473 | 3.40 (3.06-3.78) | Reference | Reference | Reference |
| Moderate WC & moderate FPG group | 1405/21 756 | 6.21 (5.89-6.54) | 1.22 (1.07-1.39) | 1.23 (1.07-1.40) | 1.23 (1.07-1.40) |
| Highest WC & high FPG group | 661/6465 | 10.10 (9.35-10.90) | 1.54 (1.31-1.82) | 1.56 (1.30-1.86) | 1.56 (1.30-1.86) |
| High WC & highest FPG group | 306/2078 | 15.42 (13.78-17.25) | 2.31 (1.93-2.77) | 1.91 (1.51-2.40) | 1.91 (1.51-2.40) |
| WHtR & FPG |  |  |  |  |  |
| Low WHtR & low FPG group | 379/10 323 | 3.45 (3.12-3.81) | Reference | Reference | Reference |
| Moderate WHtR & moderate FPG group | 1494/22 302 | 6.45 (6.13-6.78) | 1.33 (1.17-1.50) | 1.30 (1.14-1.47) | 1.24 (1.09-1.41) |
| Highest WHtR & high FPG group | 531/5065 | 10.45 (9.60-11.38) | 1.68 (1.42-1.99) | 1.60 (1.33-1.91) | 1.45 (1.21-1.74) |
| High WHtR & highest FPG group | 311/2082 | 15.68 (14.03-17.52) | 2.51 (2.10-3.00) | 2.02 (1.61-2.53) | 2.10 (1.68-2.64) |
| AVI & FPG |  |  |  |  |  |
| Low AVI & low FPG group | 552/13 199 | 3.95 (3.63-4.29) | Reference | Reference | Reference |
| Moderate AVI & moderate FPG group | 1373/20 005 | 6.61 (6.27-6.97) | 1.15 (1.03-1.29) | 1.16 (1.03-1.30) | 1.11 (0.99-1.25) |
| Highest AVI & high FPG group | 485/4509 | 10.68 (9.78-11.68) | 1.45 (1.24-1.69) | 1.46 (1.23-1.73) | 1.33 (1.11-1.58) |
| High AVI & highest FPG group | 305/2059 | 15.51 (13.86-17.35) | 2.15 (1.82-2.54) | 1.76 (1.42-2.19) | 1.84 (1.48-2.29) |
| BRI & FPG |  |  |  |  |  |
| Low BRI & low FPG group | 669/15 357 | 4.11 (3.81-4.43) | Reference | Reference | Reference |
| Moderate BRI & moderate FPG group | 1382/19 091 | 7.00 (6.64-7.38) | 1.24 (1.11-1.37) | 1.21 (1.08-1.34) | 1.15 (1.04-1.29) |
| Highest BRI & high FPG group | 352/3250 | 10.88 (9.80-12.08) | 1.52 (1.29-1.79) | 1.43 (1.20-1.71) | 1.28 (1.07-1.54) |
| High BRI & highest FPG group | 312/2074 | 15.81 (14.15-17.66) | 2.27 (1.93-2.66) | 1.83 (1.49-2.26) | 1.93 (1.56-2.38) |
| WHR & FPG |  |  |  |  |  |
| Low WHR & low FPG group | 283/7049 | 3.78 (3.37-4.25) | Reference | Reference | Reference |
| High WHR & moderate FPG group | 2112/30 554 | 6.66 (6.39-6.96) | 1.09 (0.95-1.24) | 1.07 (0.93-1.23) | 1.07 (0.93-1.22) |
| High WHR & high FPG group | 320/2169 | 15.48 (13.87-17.27) | 1.95 (1.63-2.34) | 1.58 (1.25-1.99) | 1.69 (1.34-2.13) |
| C-index &FPG |  |  |  |  |  |
| Low C-index & low FPG group | 1555/28 531 | 5.17 (4.92-5.43) | Reference | Reference | Reference |
| High C-index & moderate FPG group | 838/9039 | 9.25 (8.64-9.90) | 1.23 (1.13-1.34) | 1.22 (1.10-1.35) | 1.16 (1.05-1.27) |
| Moderate C-index & High FPG group | 322/2202 | 15.31 (13.73-17.08) | 1.93 (1.68-2.22) | 1.59 (1.32-1.91) | 1.69 (1.39-2.04) |
| ABSI &FPG |  |  |  |  |  |
| Low ABSI & low FPG group | 2015/32 554 | 5.91 (5.65-6.17) | Reference | Reference | Reference |
| High ABSI & low FPG group | 378/5006 | 7.54 (6.82-8.34) | 1.08 (0.96-1.21) | 0.97 (0.85-1.10) | 0.94 (0.83-1.06) |
| Moderate ASBI & high FPG group | 322/2212 | 15.25 (13.68-17.02) | 1.79 (1.56-2.05) | 1.42 (1.19-1.70) | 1.51 (1.25-1.82) |

Abbreviations: ABSI, a new body shape index; AVI, abdominal volume index; BRI, body roundness index; C-index, conicity index; FPG, fasting plasma glucose; WC, waist circumference; WHR, waist-to-hip ratio; WHtR, waist-to-height ratio.

^a^ Adjusted for age, sex, education background, marital status, smoking status, drinking status, physical activities, salt intake habits, LDL-C, HDL-C, ln eGFR, ln CRP, hypertension, using antihypertensive, using antidiabetic, using lipid-lowering, and additional BMI.

^b^ Adjusted for age, sex, education background, marital status, smoking status, drinking status, physical activities, salt intake habits, LDL-C, HDL-C, ln eGFR, ln CRP, hypertension, using antihypertensive, using antidiabetic, using lipid-lowering, and additional corresponding CA indices and FPG in 2006/07 survey.

^c^ Adjusted for age, sex, education background, marital status, smoking status, drinking status, physical activities, salt intake habits, LDL-C, HDL-C, ln eGFR, ln CRP, hypertension, using antihypertensive, using antidiabetic, using lipid-lowering, and additional corresponding CA indices and FPG in 2010/11 survey.

Supplementary Table S12. Adjusted Hazard Ratios (HRs) and 95% Confidence Intervals (95% CIs) of Cardiovascular Diseases According to Dual Trajectory Patterns of Various Central Adiposity Indices and Fasting Plasma Glucose Prior to Multiple Imputation of Covariates

| **Dual trajectory groups** | **Case/N** | **Incidence rate (95% CI),**  **per 1000 person-years** | **Adjusted HR (95% CI)^*^** |
| --- | --- | --- | --- |
| WC & FPG |  |  |  |
| Low WC & low FPG group | 343/9473 | 3.40 (3.06-3.78) | Reference |
| Moderate WC & moderate FPG group | 1405/21 756 | 6.21 (5.89-6.54) | 1.27 (1.12-1.43) |
| Highest WC & high FPG group | 661/6465 | 10.10 (9.35-10.90) | 1.64 (1.42-1.89) |
| High WC & highest FPG group | 306/2078 | 15.42 (13.78-17.25) | 2.36 (1.97-2.81) |
| WHtR & FPG |  |  |  |
| Low WHtR & low FPG group | 379/10 323 | 3.45 (3.12-3.81) | Reference |
| Moderate WHtR & moderate FPG group | 1494/22 302 | 6.45 (6.13-6.78) | 1.37 (1.22-1.54) |
| Highest WHtR & high FPG group | 531/5065 | 10.45 (9.60-11.38) | 1.74 (1.51-2.01) |
| High WHtR & highest FPG group | 311/2082 | 15.68 (14.03-17.52) | 2.53 (2.13-3.01) |
| AVI & FPG |  |  |  |
| Low AVI & low FPG group | 552/13 199 | 3.95 (3.63-4.29) | Reference |
| Moderate AVI & moderate FPG group | 1373/20 005 | 6.61 (6.27-6.97) | 1.21 (1.09-1.35) |
| Highest AVI & high FPG group | 485/4509 | 10.68 (9.78-11.68) | 1.55 (1.36-1.77) |
| High AVI & highest FPG group | 305/2059 | 15.51 (13.86-17.35) | 2.20 (1.87-2.59) |
| BRI & FPG |  |  |  |
| Low BRI & low FPG group | 669/15 357 | 4.11 (3.81-4.43) | Reference |
| Moderate BRI & moderate FPG group | 1382/19 091 | 7.00 (6.64-7.38) | 1.28 (1.16-1.41) |
| Highest BRI & high FPG group | 352/3250 | 10.88 (9.80-12.08) | 1.59 (1.38-1.83) |
| High BRI & highest FPG group | 312/2074 | 15.81 (14.15-17.66) | 2.29 (1.95-2.68) |
| WHR & FPG |  |  |  |
| Low WHR & low FPG group | 283/7049 | 3.78 (3.37-4.25) | Reference |
| High WHR & moderate FPG group | 2112/30 554 | 6.66 (6.39-6.96) | 1.15 (1.01-1.31) |
| High WHR & high FPG group | 320/2169 | 15.48 (13.87-17.27) | 2.04 (1.70-2.45) |
| C-index &FPG |  |  |  |
| Low C-index & low FPG group | 1555/28 531 | 5.17 (4.92-5.43) | Reference |
| High C-index & moderate FPG group | 838/9039 | 9.25 (8.64-9.90) | 1.24 (1.14-1.36) |
| Moderate C-index & High FPG group | 322/2202 | 15.31 (13.73-17.08) | 1.93 (1.68-2.23) |
| ABSI &FPG |  |  |  |
| Low ABSI & low FPG group | 2015/32 554 | 5.91 (5.65-6.17) | Reference |
| High ABSI & low FPG group | 378/5006 | 7.54 (6.82-8.34) | 1.03 (0.92-1.16) |
| Moderate ASBI & high FPG group | 322/2212 | 15.25 (13.68-17.02) | 1.77 (1.54-2.04) |

Abbreviations: ABSI, a new body shape index; AVI, abdominal volume index; BRI, body roundness index; C-index, conicity index; CVD, cardiovascular disease; FPG, fasting plasma glucose; WC, waist circumference; WHR, waist-to-hip ratio; WHtR, waist-to-height ratio.

^*^ Adjusted for age, sex, education background, marital status, smoking status, drinking status, physical activities, salt intake habits, LDL-C, HDL-C, ln eGFR, ln CRP, hypertension, using antihypertensive, using antidiabetic, and using lipid-lowering.

Supplementary Table S13. Adjusted Hazard Ratios (HRs) and 95% Confidence Intervals (95% CIs) of Cardiovascular Diseases According to Dual trajectory Patterns of Various Central Adiposity Indices and Fasting Plasma Glucose After Excluding Medication Usage

| **Dual trajectory groups** | **Case/N** | **Incidence rate (95% CI),**  **per 1000 person-years** | **Adjusted HR (95% CI)^*^** |
| --- | --- | --- | --- |
| WC & FPG |  |  |  |
| Low WC & low FPG group | 298/9025 | 3.10 (2.76-3.47) | Reference |
| Moderate WC & moderate FPG group | 1172/19 486 | 5.75 (5.43-6.09) | 1.30 (1.14-1.48) |
| Highest WC & high FPG group | 490/5163 | 9.27 (8.48-10.12) | 1.73 (1.49-2.01) |
| High WC & highest FPG group | 176/1204 | 15.34 (13.24-17.79) | 2.70 (2.23-3.27) |
| WHtR & FPG |  |  |  |
| Low WHtR & low FPG group | 337/9869 | 3.20 (2.87-3.56) | Reference |
| Moderate WHtR & moderate FPG group | 1234/19 876 | 5.94 (5.62-6.28) | 1.38 (1.22-1.56) |
| Highest WHtR & high FPG group | 383/3923 | 9.63 (8.71-10.64) | 1.81 (1.55-2.11) |
| High WHtR & highest FPG group | 182/1210 | 15.85 (13.71-18.33) | 2.88 (2.39-3.46) |
| AVI & FPG |  |  |  |
| Low AVI & low FPG group | 478/12481 | 3.61 (3.30-3.94) | Reference |
| Moderate AVI & moderate FPG group | 1130/17 656 | 6.14 (5.79-6.50) | 1.25 (1.12-1.39) |
| Highest AVI & high FPG group | 352/3550 | 9.72 (8.75-10.79) | 1.63 (1.41-1.89) |
| High AVI & highest FPG group | 176/1191 | 15.52 (13.39-17.99) | 2.53 (2.12-3.02) |
| BRI & FPG |  |  |  |
| Low BRI & low FPG group | 589/14 548 | 3.81 (3.51-4.13) | Reference |
| Moderate BRI & moderate FPG group | 1119/16 668 | 6.45 (6.08-6.84) | 1.30 (1.18-1.44) |
| Highest BRI & high FPG group | 245/2458 | 9.87 (8.71-11.19) | 1.65 (1.41-1.93) |
| High BRI & highest FPG group | 183/1204 | 16.02 (13.86-18.52) | 2.62 (2.21-3.10) |
| WHR & FPG |  |  |  |
| Low WHR & low FPG group | 241/6578 | 3.44 (3.03-3.90) | Reference |
| High WHR & moderate FPG group | 1710/27 040 | 6.06 (5.78-6.35) | 1.15 (1.00-1.32) |
| High WHR & high FPG group | 185/1260 | 15.44 (13.37-17.83) | 2.30 (1.89-2.80) |
| C-index &FPG |  |  |  |
| Low C-index & low FPG group | 1306/26 039 | 4.74 (4.49-5.00) | Reference |
| High C-index & moderate FPG group | 642/7552 | 8.40 (7.78-9.08) | 1.24 (1.13-1.37) |
| Moderate C-index & High FPG group | 188/1287 | 15.33 (13.29-17.69) | 2.17 (1.86-2.53) |
| ABSI &FPG |  |  |  |
| Low ABSI & low FPG group | 1651/29 241 | 5.36 (5.11-5.62) | Reference |
| High ABSI & low FPG group | 299/4351 | 6.80 (6.07-7.62) | 1.02 (0.90-1.16) |
| Moderate ASBI & high FPG group | 186/1286 | 15.16 (13.13-17.51) | 1.99 (1.71-2.32) |

Abbreviations: ABSI, a new body shape index; AVI, abdominal volume index; BRI, body roundness index; C-index, conicity index; CVD, cardiovascular disease; FPG, fasting plasma glucose; WC, waist circumference; WHR, waist-to-hip ratio; WHtR, waist-to-height ratio.

^*^ Adjusted for age, sex, education background, marital status, smoking status, drinking status, physical activities, salt intake habits, LDL-C, HDL-C, ln eGFR, ln CRP, and hypertension.

Supplementary Table S14. Adjusted Hazard Ratios (HRs) and 95% Confidence Intervals (95% CIs) of Cardiovascular Diseases According to Dual trajectory Patterns of Various Central Adiposity Indices and Fasting Plasma Glucose After Excluding Outcomes Within the Initial 2 Years of Follow-Up

| **Dual trajectory groups** | **Case/N** | **Incidence rate (95% CI),**  **per 1000 person-years** | **Adjusted HR (95% CI)^*^** |
| --- | --- | --- | --- |
| WC & FPG | 312/9375 | 3.10 (2.77-3.46) |  |
| Low WC & low FPG group | 1250/21 415 | 5.53 (5.23-5.85) | Reference |
| Moderate WC & moderate FPG group | 586/6329 | 8.97 (8.27-9.73) | 1.24 (1.09-1.40) |
| Highest WC & high FPG group | 259/2003 | 13.10 (11.60-14.80) | 1.62 (1.40-1.88) |
| High WC & highest FPG group | 312/9375 | 3.10 (2.77-3.46) | 2.28 (1.90-2.74) |
| WHtR & FPG |  |  |  |
| Low WHtR & low FPG group | 342/10 214 | 3.11 (2.80-3.46) | Reference |
| Moderate WHtR & moderate FPG group | 1337/21 958 | 5.78 (5.48-6.10) | 1.36 (1.21-1.54) |
| Highest WHtR & high FPG group | 464/4942 | 9.15 (8.36-10.02) | 1.75 (1.51-2.02) |
| High WHtR & highest FPG group | 264/2008 | 13.36 (11.84-15.08) | 2.48 (2.07-2.97) |
| AVI & FPG |  |  |  |
| Low AVI & low FPG group | 494/13 049 | 3.54 (3.24-3.86) | Reference |
| Moderate AVI & moderate FPG group | 1224/19 682 | 5.91 (5.58-6.25) | 1.20 (1.08-1.34) |
| Highest AVI & high FPG group | 431/4407 | 9.52 (8.66-10.46) | 1.58 (1.38-1.82) |
| High AVI & highest FPG group | 258/1984 | 13.17 (11.66-14.88) | 2.16 (1.82-2.57) |
| BRI & FPG |  |  |  |
| Low BRI & low FPG group | 600/15 179 | 3.69 (3.40-4.00) | Reference |
| Moderate BRI & moderate FPG group | 1236/18 775 | 6.27 (5.93-6.63) | 1.29 (1.17-1.42) |
| Highest BRI & high FPG group | 306/3170 | 9.48 (8.48-10.61) | 1.62 (1.40-1.87) |
| High BRI & highest FPG group | 265/1998 | 13.48 (11.95-15.20) | 2.27 (1.93-2.68) |
| WHR & FPG |  |  |  |
| Low WHR & low FPG group | 238/6975 | 3.18 (2.80-3.62) | Reference |
| High WHR & moderate FPG group | 1897/30 059 | 6.00 (5.73-6.27) | 1.24 (1.08-1.43) |
| High WHR & high FPG group | 272/2088 | 13.21 (11.73-14.88) | 2.16 (1.78-2.62) |
| C-index &FPG |  |  |  |
| Low C-index & low FPG group | 1395/28 186 | 4.64 (4.41-4.89) | Reference |
| High C-index & moderate FPG group | 738/8814 | 8.16 (7.60-8.78) | 1.24 (1.13-1.36) |
| Moderate C-index & High FPG group | 274/2122 | 13.08 (11.62-14.73) | 1.90 (1.64-2.21) |
| ABSI &FPG |  |  |  |
| Low ABSI & low FPG group | 1796/32 110 | 5.27 (5.03-5.52) | Reference |
| High ABSI & low FPG group | 337/4882 | 6.74 (6.06-7.50) | 1.05 (0.93-1.18) |
| Moderate ASBI & high FPG group | 274/2130 | 13.03 (11.58-14.67) | 1.75 (1.51-2.03) |

Abbreviations: ABSI, a new body shape index; AVI, abdominal volume index; BRI, body roundness index; C-index, conicity index; FPG, fasting plasma glucose; WC, waist circumference; WHR, waist-to-hip ratio; WHtR, waist-to-height ratio.

^*^ Adjusted for age, sex, education background, marital status, smoking status, drinking status, physical activities, salt intake habits, LDL-C, HDL-C, ln eGFR, ln CRP, hypertension, using antihypertensive, using antidiabetic, and using lipid-lowering.

Supplementary Figure S1. Kaplan-Meier Curve of Cardiovascular Disease Incidence Rate by the Time Course of Dual trajectory of Waist Circumference and Fasting Plasma Glucose


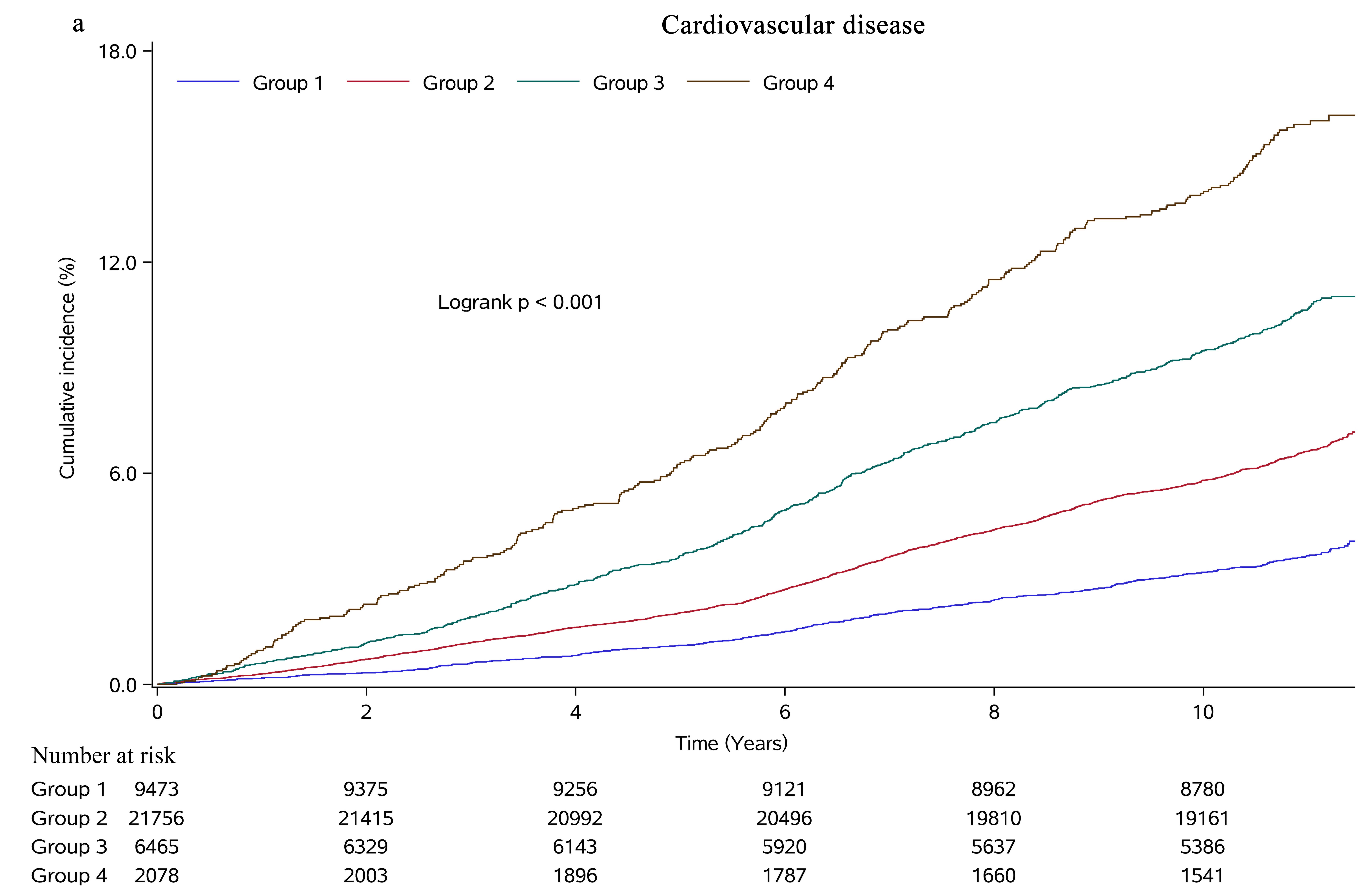


Group 1: Low WC & low FPG group; Group 2: Moderate WC & moderate FPG group; Group 3: Highest WC & high FPG group; Group 4: High WC & highest FPG group.

Supplementary Figure S2. Kaplan-Meier Curve of Cardiovascular Disease Incidence Rate by the Time Course of Dual trajectory of Waist-Height Ratio and Fasting Plasma Glucose


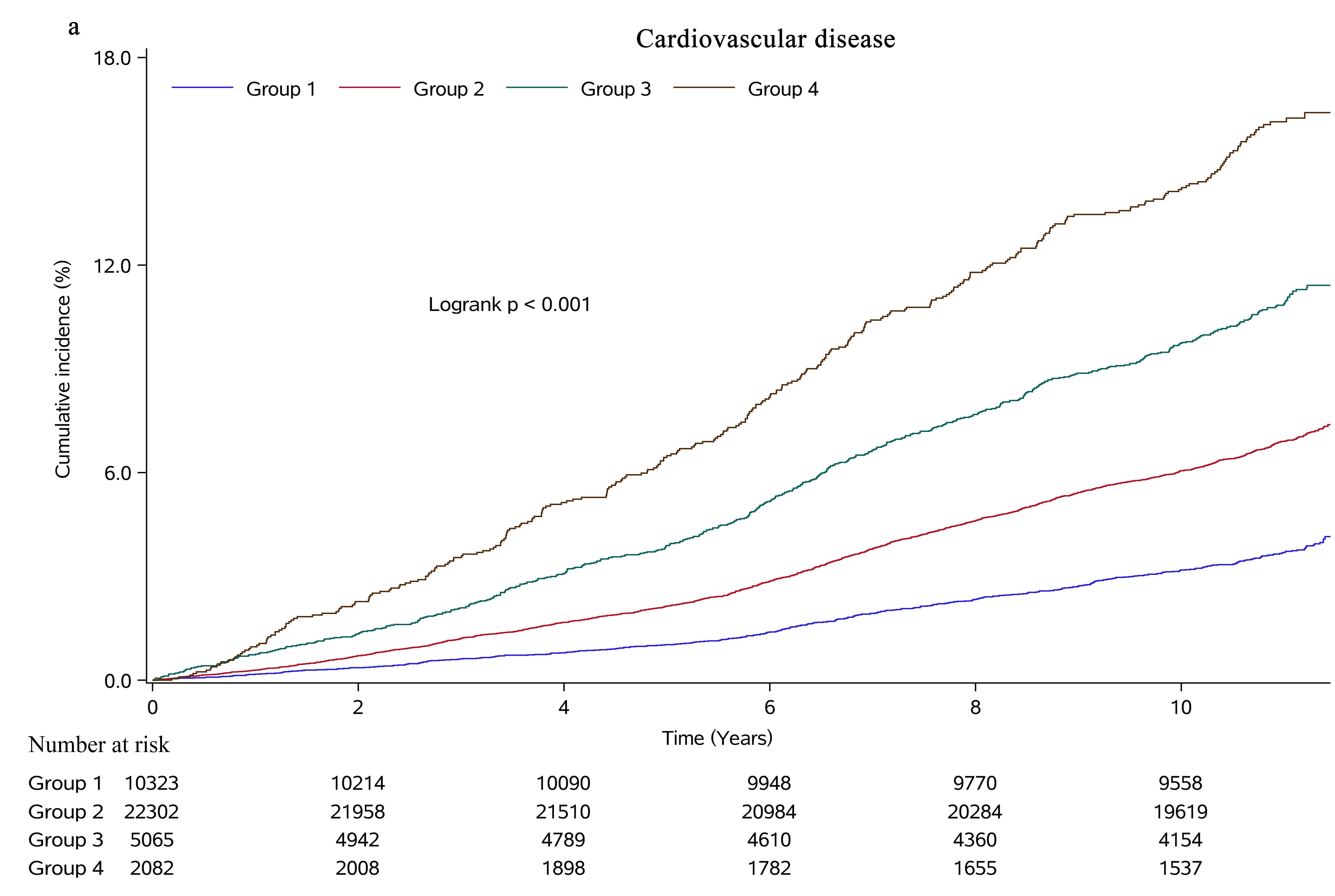


Group 1: Low WHtR & low FPG group; Group 2: Moderate WHtR & moderate FPG group; Group 3: Highest WHtR & high FPG group; Group4: High WHtR & highest FPG group.

Supplementary Figure S3. Kaplan-Meier Curve of Cardiovascular Disease Incidence Rate by the Time Course of Dual trajectory of Abdominal Volume Index and Fasting Plasma Glucose


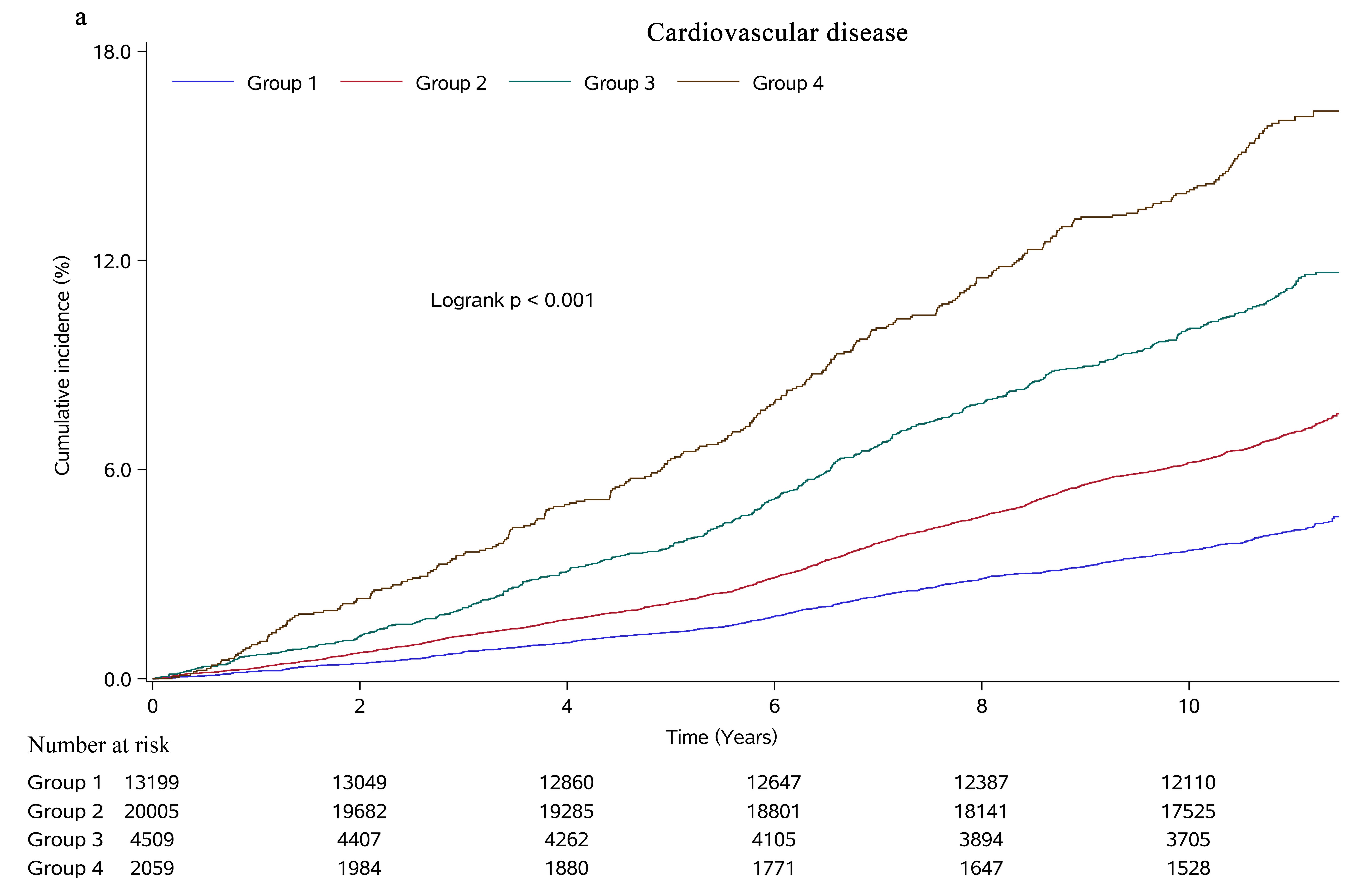


Group 1: Low AVI & low FPG group; Group 2: Moderate AVI & moderate FPG group; Group 3: Highest AVI & high FPG group; Group 4: High AVI & highest FPG group.

Supplementary Figure S4. Kaplan-Meier Curve of Cardiovascular Disease Incidence Rate by the Time Course of Dual trajectory of Body Roundness Index and Fasting Plasma Glucose


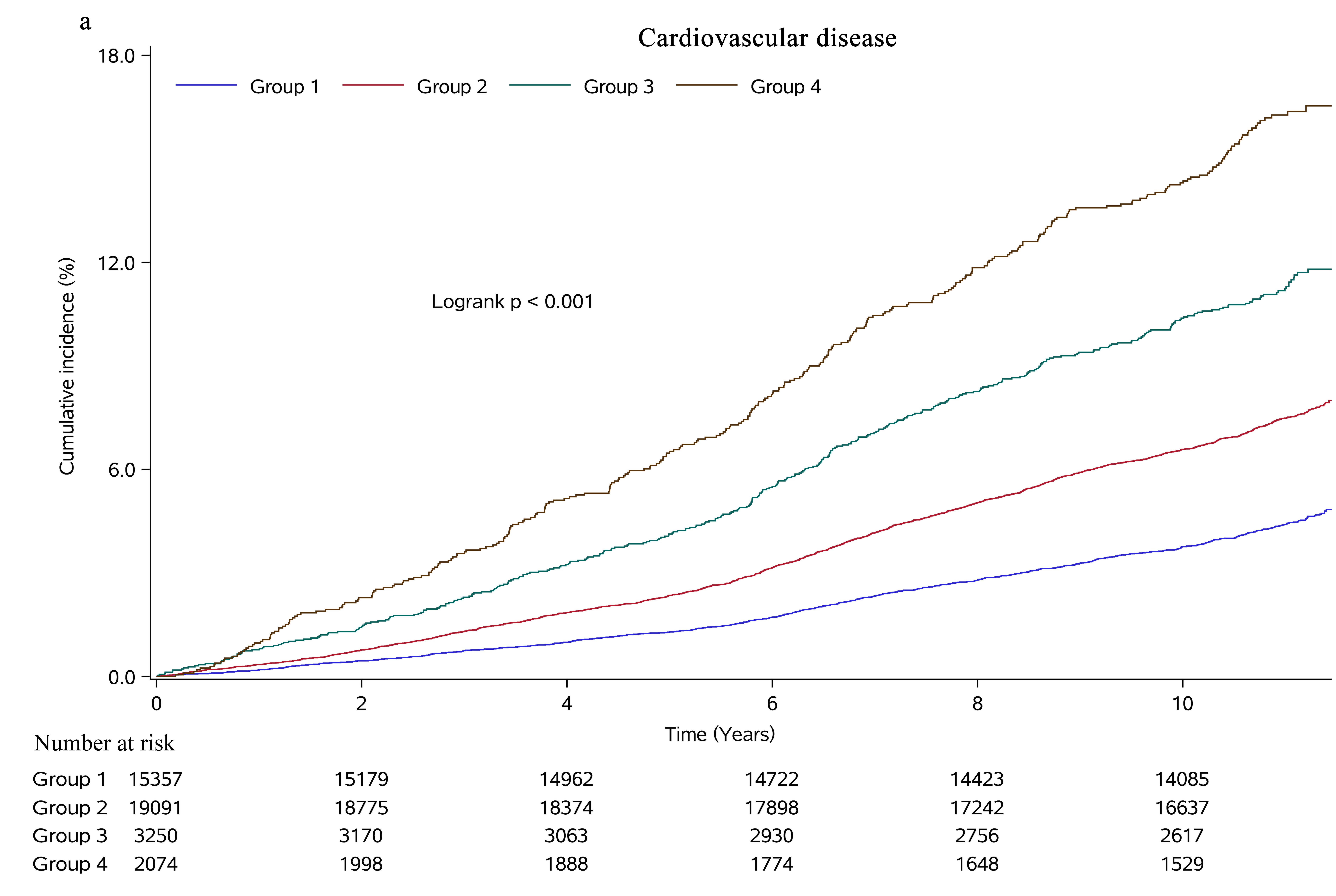


Group 1: Low BRI & low FPG group; Group 2: Moderate BRI & moderate FPG group; Group 3: Highest BRI & high FPG group; Group 4: High BRI & highest FPG group.

Supplementary Figure S5. Kaplan-Meier Curve of Cardiovascular Disease Incidence Rate by the Time Course of Dual trajectory of Waist-Hip Ratio and Fasting Plasma Glucose


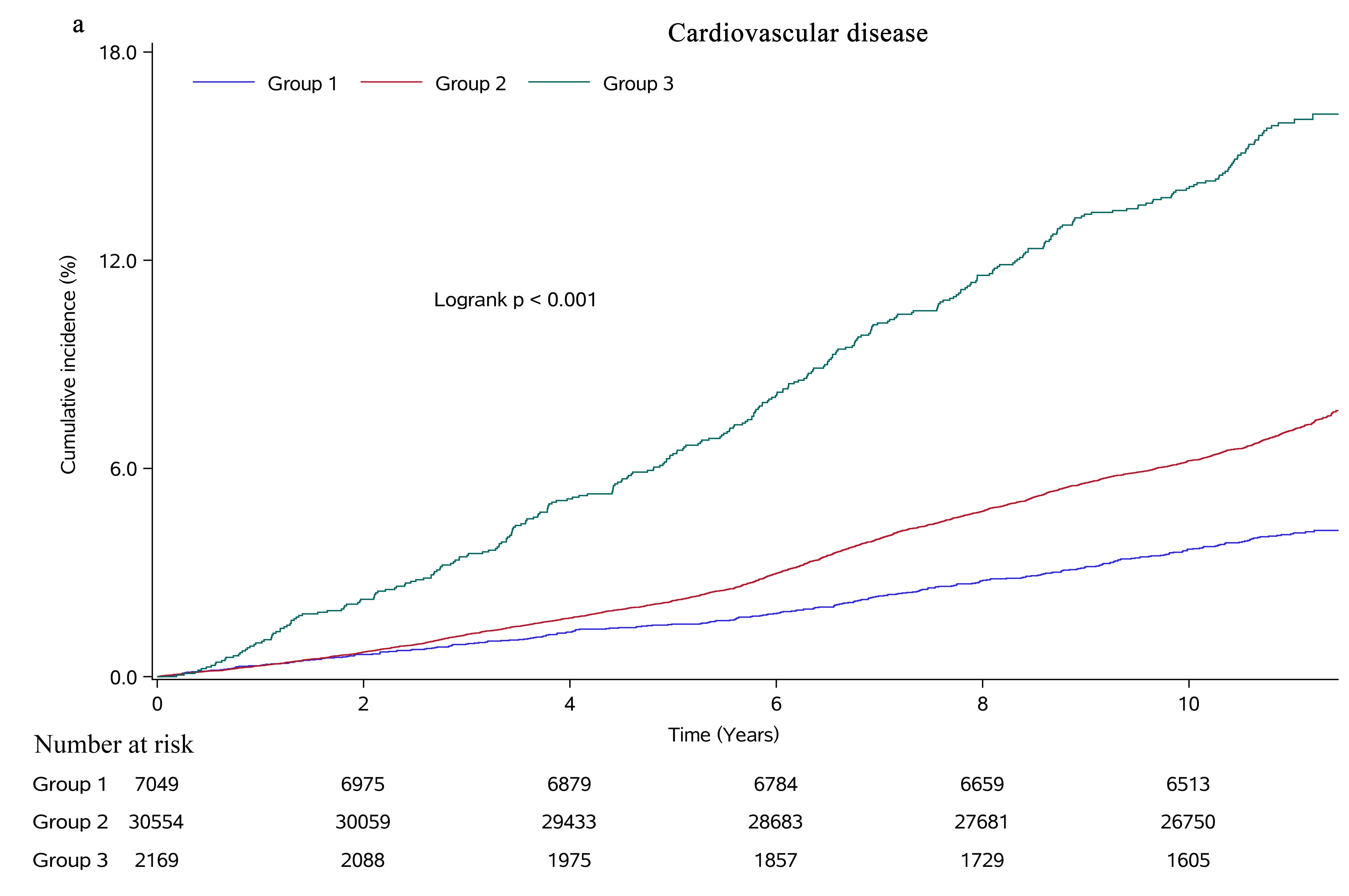


Group 1: Low WHR & low FPG group; Group 2: High WHR & moderate FPG group; Group 3: High WHR & high FPG group.

Supplementary Figure S6. Kaplan-Meier Curve of Cardiovascular Disease Incidence Rate by The Time Course of Dual trajectory of Conicity Index and Fasting Plasma Glucose


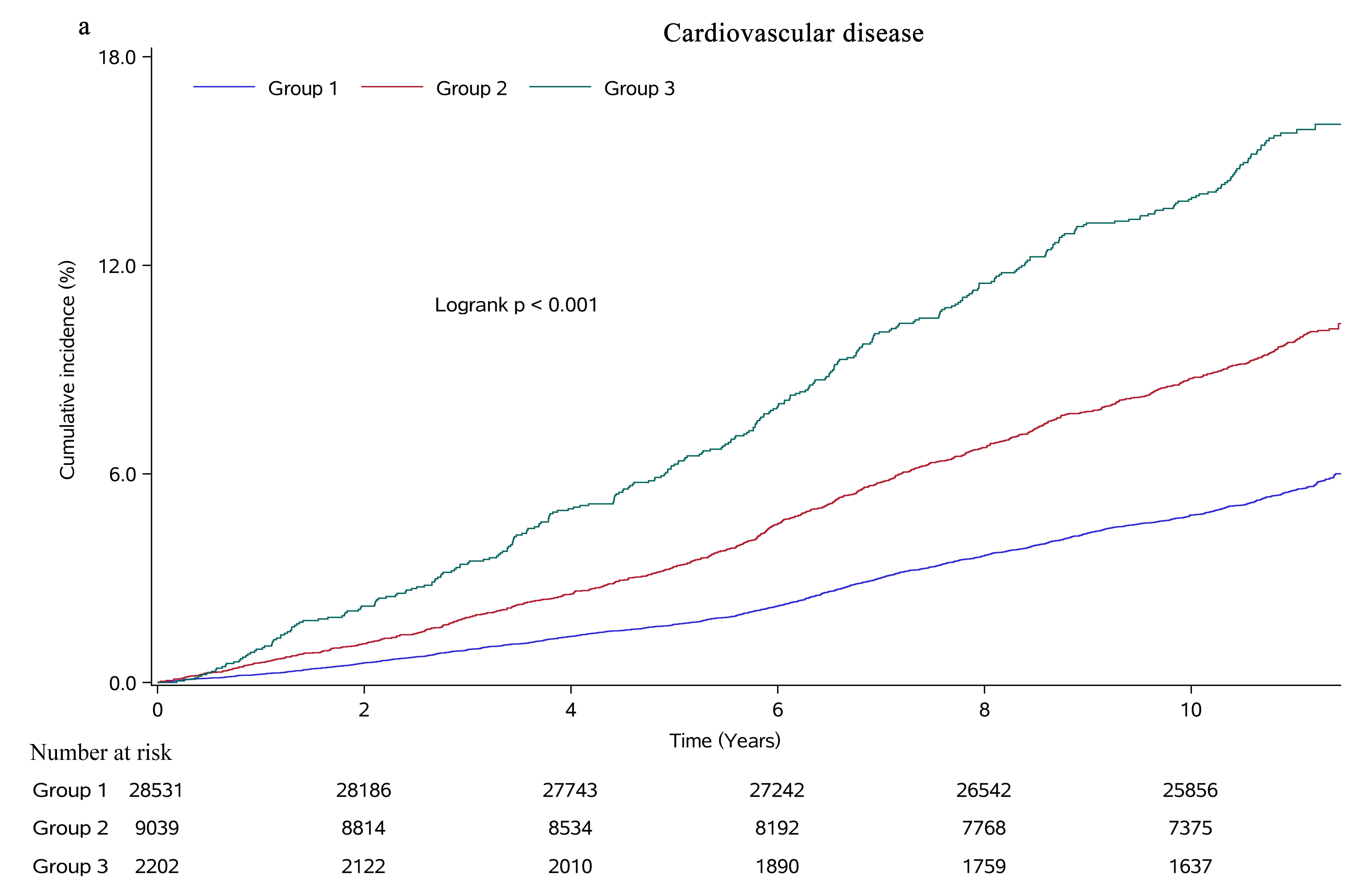


Group 1: Low C-index & low FPG group; Group 2: High C-index & moderate FPG group; Group 3: Moderate C-index & High FPG group.

Supplementary Figure S7. Kaplan-Meier Curve of Cardiovascular Disease Incidence Rate by the Time Course of Dual trajectory of A New Body Shape Index and Fasting Plasma Glucose


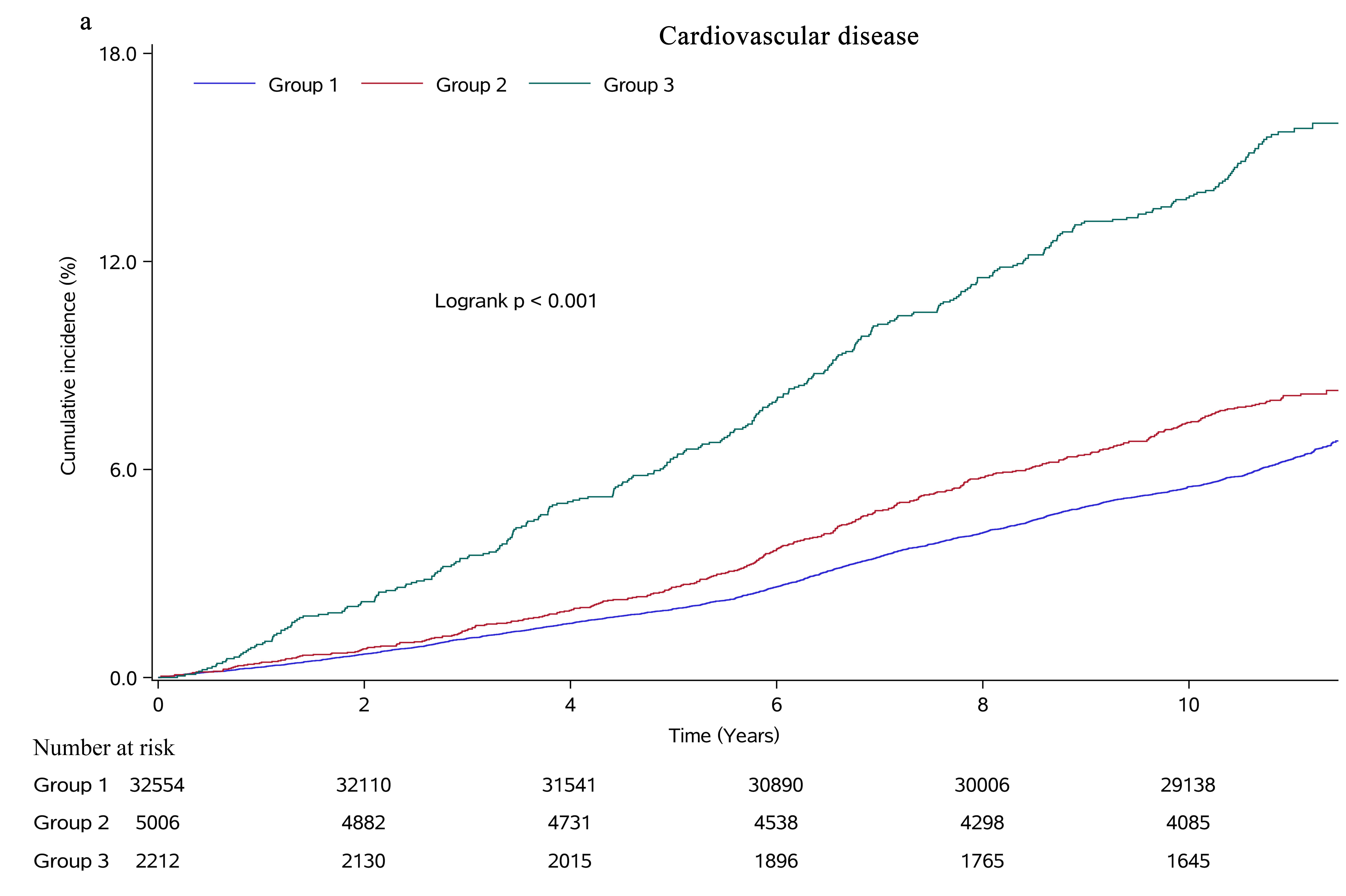


Group 1: Low ABSI & low FPG group; Group 2: High ABSI & low FPG group; Group 3: Moderate ASBI & high FPG group.
