# Supplementary material for: Predictive Capability of Dual Trajectories of Central Adiposity Indices Combined With Glucose for Cardiovascular Diseases
Source: J Diabetes. 2025 Apr 25;17(4):e70081. doi: 10.1111/1753-0407.70081 (PMC12022674; doi:10.1111/1753-0407.70081)
Supplement: Supplementary file 2 — Data S2. Supporting Information. [file JDB-17-e70081-s002.docx]

Supplemental 2

**Data Sharing Statement**

**Data available:** Yes

**Data types:** Complete de‐identified patient data set

**How to access data:** Data will be available upon reasonable request to the corresponding author(Shouling Wu)

**When available:** One year after publication

**Supporting Documents**

**Document types:** None

**Additional Information**

**Who can access the data:** researchers whose proposed use of the data has been approved

**Types of analyses:** None

**Mechanisms of data availability:** after approval of a proposal

**Any additional restrictions:** China law, law of participating centers and regulations of respective ethics committees.
